# Unravelling drought stress adaptation in sugarcane interspecific hybrids: A multi-level analysis

**DOI:** 10.1371/journal.pone.0338698

**Published:** 2025-12-12

**Authors:** Lakshmi Kasirajan, Sheelamary Sebastiar, Ramvannish Muthukumar, Rachel Lissy Vargheese, Karpagam Elumalai, Keerthana Kamaraj, Rabisha Valiyaparambth, Suganya Angannan, Annadurai Ayyadurai, Gomathi Raju, Selvi Athiappan, Devakumar Krishnamoorthy, Gayathri Kanagavel, Nandini Karthikeyan, Palanivel Gopal Santhosh Kumar, Indusha Yazhini Sankararaj

**Affiliations:** 1 Division of Crop Improvement, ICAR Sugarcane Breeding Institute, Coimbatore, Tamil Nadu, India; 2 Division of Crop Production, ICAR Sugarcane Breeding Institute, Coimbatore, Tamil Nadu, India; 3 Karunya University, Coimbatore, Tamil Nadu, India; Nuclear Science and Technology Research Institute, IRAN, ISLAMIC REPUBLIC OF

## Abstract

Drought stress, a major abiotic stress, severely limits sugarcane productivity through adversely altered cellular processes. Identifying key traits linked to physiological and biochemical responses during water stress and recovery is vital for screening and breeding drought stress-tolerant varieties. The present study assessed 18 sugarcane genotypes based on morpho-physiological, biochemical, and molecular changes during drought stress and at stress recovery in a complete randomized block design. Cane height, number of internodes, Soil Plant Analytical Development (SPAD) index, Leaf Area Index (LAI), Relative Water Content (RWC), Nitrate Reductase (NR), and proline were positively correlated to drought stress. Among the genotypes studied, AS-04-1687, AS-04-635, Co 86032, Co 85019, CoM 0265, AS-04-2097, and AS-04-245 were identified as potential clones with high potential for drought stress tolerance in sugarcane. Expression profiling of ten stress-induced genes, namely NAC, MYB59, peroxidase, GST (glutathione transferase), GLY I (glyoxylase I), GLY III (glyoxylase III), RAB (response to abscisic acid), CesA3 (cellulose synthase), heat shock proteins, and stress-related proteins in sugarcane stem tissues under drought-stressed conditions revealed significant differences between tolerant and susceptible genotypes. The interspecific hybrid, AS-04-1687, outperformed for all the genes under drought stress conditions and could be a potential donor in further drought tolerance breeding programmes. We have also cloned and characterised drought stress-induced transcription factor MYB59 (a negative regulator of Ca^+^ signalling), which could be used in genetic engineering programmes for the development of drought stress-tolerant genotypes.

## Introduction

Sugarcane is cultivated globally for sucrose and bioenergy. India is the second largest producer of sugar, with a 20% contribution toward the global production [1]. The lengthy duration of the crop makes it vulnerable to natural constraints [2], and its yield is largely affected by climate change, altered rainfall patterns, and fluctuating environmental conditions [3]. Abiotic stress factors, *viz*., drought stress, salinity, waterlogging, excess light, heat/cold stress, and nutrient deficiency, lead to impaired growth and development [4]. Amongst these, drought stress is considered a major limiting factor with large destructive effects on cane yield [5,6] and a maximum productivity loss of 60% [7]. In India, approximately 2.97 lakh ha of land is exposed to drought stress with low productivity [6]. Drought stress occurs when annual rainfall is below normal, the groundwater level is depleted, and evapotranspiration exceeds water uptake [8]. Sugarcane is a water-intensive crop [9], and the summer dry period is usually accompanied by the formative phase of the crop. It impacts tiller production, cane weight, cane height, and yield, as well as juice quality [10].

Drought stress tolerance in sugarcane has not been completely studied due to the complexity at both the genome level and in plant defence responses. Morpho-physiological traits possessing high heritability, combined with a positive correlation to high yield and the ability to be measured at early stages of development, have gained increased attention for early selection in sugarcane drought stress tolerance evaluation programmes [11,12]. Studies of Silva et al. (2011 and 2012) [13–15] reported that genotypes under drought stress with high chlorophyll fluorescence ratio (Fv/Fm), Soil Plant Analytical Development (SPAD) index, leaf relative water content (RWC), transpiration rate, along with lower leaf canopy temperature indicated high tolerance. Various studies highlight the SPAD index as a reliable and fast screening method to explain nitrogen status and plant water use efficiency under drought stress [11,16,17]. Proline accumulation is studied as the most efficient biochemical marker, and high concentration is linked to higher tolerance of abiotic stress [18]. Li et al. (2021) [19] studied physiological changes and transcriptome profiling in *S. spontaneum* L. leaf under drought stress and recovery conditions. He reported that high accumulation of osmo-protectant (proline), plant hormones (abscisic acid, SA and indole acetic acid), and antioxidant activity (catalase, superoxide dismutase, ascorbate peroxidase, and glutathione reductase), along with enriched plant protein kinases and hormone signal transduction pathways, are responsible for drought stress tolerance. Reduction of cane yield under drought stress conditions and stay-green traits were investigated by Wirojsirasak et al. (2024) [20], and they identified high Fv/Fm, SPAD, RWC, and lower leaf rolling and leaf drying as selection criteria to categorise genotypes with high yield potential and low reduction under drought stress conditions in sugarcane.

At the molecular level, stress signalling pathways are activated through improved ion transport, stomatal regulation, transcription factors and phytohormones for enhanced drought stress [21–24]. Transcriptomic studies were used to evaluate genes described with environmental stress avoidance, *viz*., leaf gas exchange, leaf abscission, maturation and cellulose and lignin biosynthesis, to study gene annotation and its functional changes under stress to improve productivity [25–28]. Heringer et al. (2017) [29] observed that upregulation of ERD4 (early responsive dehydration protein-4) and P5CS (pyrroline-5-carboxylase synthase) were linked to drought stress tolerance. Similarly, overexpression of EaDREB2 transferred from E. *arundinaceus* [30] also reported the same. In an experiment by McQualter (2007) [31], up-regulation of ScMYB59 (sugar cane transcription factor gene-Myeloblastosis) activated the abscisic acid signalling pathway, thereby enhancing stress tolerance.

In this view, the present study has evaluated drought stress-induced responses of 18 sugarcane genotypes comprising four ISH (interspecific hybrid) and fourteen Co canes at field conditions for three consecutive field trials at different locations, and their molecular tolerance mechanisms involved drought stress resilience. We have also identified and cloned the most promising transcription factor, MYB59, for the tolerant genotype, which might be used for the genetic transformation of sugarcane for drought stress tolerance.

## Materials and methods

### Plant material and experimental design

Fourteen Co canes (sugarcane commercial hybrids including drought stress susceptible and tolerant clones) and four ISH clones (sugarcane interspecific hybrids) (S1 Table) were evaluated at the ICAR-Sugarcane Breeding Institute (11^◦^0’34” N, 76^◦^55’2” E), Coimbatore, Tamil Nadu, India, for drought stress tolerance. The experiment was conducted in a complete randomized block design (CRBD) with two replications. Two-budded setts for all genotypes were planted in a row of 6.0 m length with 1.20 m spacing between rows during 2023, in a total plot area of 576 m^2^. The experimental block was divided into control and drought-stressed treatments. Fertiliser dosage and all recommended cultivation practices were uniformly followed up to 90 days after planting (DAP) to establish a disease-free, healthy crop with uniform growth, and the details of the soil nutrients are given in S2 Table. The drought stress was artificially induced by withholding irrigation for 30 days (May-June), and the average rainfall data recorded during the period was 0.4 mm (S3 Table), whereas the control plot received normal irrigation. This period also coincided with the formative/tillering phase of the crop, which is identified to be the sensitive phase of development in sugarcane [32]. During the recovery period, both the control and drought plots were irrigated up to field capacity once a week.

### Evaluation of morpho-physiological traits during drought stress

Morphological, physiological and biochemical parameters were studied at 120 DAP, the formative growth phase (F) after imposing drought stress. All traits were evaluated in both the control and drought stress treatments. Tiller count (TC), cane thickness (CT^F^) (cm) and cane height (CH^F^) (cm) were recorded at 120 DAP, the formative growth phase (during the drought stress). Number of millable cane (NMC) (t/ha), cane thickness (CT^M^) (cm), cane height (CH^M^) (cm) and SCW (kg) were recorded at 300 DAP during the maturity growth phase (after stress recovery). The cane thickness (CT) was measured using a digital vernier calliper (Mitutoyo, Kawasaki, Japan) at the middle portion of three randomly selected canes in each replication and both treatments. Three selected canes were cut close to ground level, and after removing the leaf tops, the cane height was measured using a measuring scale. Single cane weight (SCW) (kg) was measured at 300 DAP; three randomly chosen canes were weighed in an electronic platform balance to record three cane weights, and then SCW was calculated. The number of internodes (No. of IN) was studied at 120 DAP (during stress). Cane yield (t/ha) was recorded by weighing plot yield at 300 DAP in both control and treatment, which was converted to yield per hectare. The yield was calculated by multiplying the number of millable canes by the single cane weight [33]. Sugarcane juice quality was analysed as Brix% (total soluble sugars) and pol% (sucrose content) using a Brix hydrometer and polarimeter, respectively, according to the method given by Meade et al. (1977) [34]. The CCS% (commercial cane sugar) was also determined based on the formula given below [34].


CCS%=pol%×1.022/brix%×0.292 and CCS yield (t/ha)=CCS%×cane yield/100

Five physiological traits, *viz*., SPAD reading (soil plant analysis development), leaf area index (LAI), relative water content (RWC), chlorophyll fluorescence (*Fv/Fm*) and canopy temperature, were studied at 120 DAP in control and drought stress. The non-destructive chlorophyll estimation was measured using a SPAD meter (Leaf, Wilmington, Delaware, NC, USA). Measurements were taken between 09.00 am and 11.00 am to record transmission of red light and infrared light at 660 nm and 940 nm, respectively, to compute leaf chlorophyll content. Leaf surface area was calculated by LA = LBK (cm^2^) [35], L = leaf length, B = maximum leaf breadth, and K = constant (0.75 based on regression analysis) and then leaf area index (LAI) (m^2^m^-2^) was computed by following the method of Watson, 1958 [36] RWC (%) was estimated following the procedure given by Barrs and Weatherley, 1962 [37]. Leaf discs of uniform sizes (2.5 cm^2^) from the first fully expanded leaf were weighed (fresh weight) and soaked in deionised water for 4 hr. After hydration, turgid weight (TW) was recorded, and then completely dried (at 80^°^C) to measure dry weight (DW). RWC (%) = [(FW-DW)/ (TW-DW)] ×100.

The canopy temperature (^°^C) was recorded with a thermal imaging infrared camera (FLIR E6, image processor- FLIR software-FLIR Tools version 5.1.15036.1001) between 11:30 am and 12:00 pm. The camera was held at 30^0^ angle to crop, and images were captured in Thermal MSX® mode (FLIR Systems, Wilsonville, OR, USA) and saved in JPG format. Chlorophyll fluorescence (*Fv/Fm*) [fluorescence emissions emanating from chlorophyll a (photosystem II)] is highly sensitive to stress and estimates the inner mechanisms of the light reaction of photosynthesis [38]. *Fv/Fm* was measured using a chlorophyll fluorometer (model OS30p, Opti-Sciences, Hudson, NH, USA). The leaves were given dark adaptation for 15 min with leaf clips (Opti Sciences), and then *Fv/Fm* was quantified by passing a saturating light: *Fv/Fm* = *Fm* – *Fo/Fm*, where *Fm* = maximal fluorescence, *Fo* = minimal fluorescence, and *Fv* = variable fluorescence of photosystem II [39].

### Estimation of biochemical parameters during drought stress

Nitrate reductase activity (NRase) and proline content were estimated during the water stress period at 120 DAP in both the control and drought stress plots. NRase activity in the leaf was estimated following the method of Hageman and Hucklesby (1971) [40]. Leaf discs of 4.0 mm^2^ (1.0 g) were suspended in incubation buffer containing 0.1 M potassium phosphate buffer (pH 7.5), 0.1 M KNO_3_ and 2.0 mM NADHNa_2_ and vacuum infiltrated for 20 min and subsequently incubated in the dark at 37°C for 1 hr. Nitrite was estimated by the method of Evans and Nason (1953) [41]. To 0.2 ml of the aliquot, 1.0% sulphanilamide in 1N-HCl and 0.025% N-(1-naphthyl)-ethylene diammonium dichloride (NEDD) were added, and the absorbance of the pink colouration formed due to diazotisation was measured at 540 nm with a UV-VIS spectrophotometer. The calibration curve was prepared using potassium nitrite solution, and enzyme activity was expressed as μmole NO_2_ g^-1^FW.

Proline content was quantified as described by Bates et al. (1973) [42]. Leaf samples (0.5 g) were homogenized with 3% (w/v) sulphosalicylic acid and centrifuged at 10,000 rpm, 4^°^C. Supernatant of 2.0 ml was added with 2.0 ml of glacial acetic acid, 2.0 ml of acid ninhydrin and 20.0 ml of 6M phosphoric acid and incubated at 100°C for 1 hr, cooled, and then the reaction mixture was extracted with 5.0 ml toluene and vigorously mixed using a vortex mixer. The absorbance of chromophore-containing toluene was measured at 520 nm with toluene as a blank. The proline content was estimated from the standard curve of D-proline and expressed as µmol g^−1^ FW.

### Statistical analyses

Analysis of variance (ANOVA) at a significant level of P < 0.05 and descriptive statistics were performed using GRAPES, an online R-based tool [43]. The R package corrplot was used to visualise the correlation matrix plot, and further, two R packages, FactoMineR and factoextra, were used to identify the principal components contributing to the variability by performing principal component analysis (PCA). Descriptive statistics were presented in boxplots using the statistical software R [44] version 4.1.1 using the ggplot2 package. The dendrogram was constructed using the hierarchical clustering method with average linkage, where the distance between the clusters was measured using the Euclidean method.

### Molecular analysis of sugarcane during drought stress

#### Plant material, total RNA isolation and cDNA conversion.

Six genotypes, *viz*., AS-04-245, AS-04-635, AS-04-1687, AS-04-2097, Co 85019 and Co 775 in both control and drought stress, were studied for gene expression at 120 DAP during drought stress. Total RNA was extracted from 2–2.5 g of stem tissues, which were ground to a fine powder in liquid nitrogen using a pestle and mortar. The pulverized tissue was transferred to a 50 ml tube and homogenized with 5 ml of Trizol (Life Technologies) per gram of tissue, according to the manufacturer’s instructions. RNA pellets were resuspended in 20 μl of warm diethyl pyrocarbonate-treated water, followed by treatment with RNase-free DNase to remove the DNA contamination. Finally, RNA samples were loaded on a 1.0% agarose gel for quality check. First-strand cDNA synthesis was performed with an oligo dT primer and M-MuLV reverse transcriptase enzyme (cDNA kit, Thermo Scientific), which was used as a template for PCR amplification.

#### Quantitative real-time PCR.

The qRT-PCR (quantitative Real-Time PCR) was done for analysing the expression of 10 genes induced during drought stress *viz*., N-acetyl cysteine (NAC), myeloblastosis (MYB59), peroxidase (POD), glutathione-s-transferase (GST), Glyoxylase I (GLY I), Glyoxylase III (GLY III), responsive to abscisic acid (RAB), cellulose synthase (CesA3), heat shock proteins (HSP) and stress-related proteins. Gene-specific primers were designed utilizing Integrated DNA Technologies [45] software with reference to gene sequences of sugarcane species and related genera from the National Centre for Biotechnology Information [46]. Gene expression was analysed with the Rotor-gene Q PCR machine of Qiagen with a reaction mixture containing template cDNA, primer and TB Green® Premix Ex Taq™ II (Tli RNaseH Plus) (TaKaRa). Reaction conditions with denaturation (95°C) and combined annealing/extension were performed in the range of 55–60°C for different primers at 5 sec for 40 cycles. For every sample, two technical replications were done, and 25S rRNA was used as an endogenous control. The specificity of the reaction was checked using melting curve analysis. The threshold cycle (Ct) was calculated with the equation of 2^^-(∆∆CT)^ [47], and fold change for every gene was computed.

#### Cloning of drought-induced transcription factor MYB59.

The nucleotide sequence of MYB59 from the sugarcane-related plant species was obtained from the GenBank database of the National Centre for Biotechnology Information (NCBI) website [46]. The primers for the coding sequence of MYB59 were designed using IDT software. PCR reactions were carried out in a final volume of 20 µl in an Eppendorf thermocycler containing 0.9 µl cDNA, 20–30 pmol of each forward and reverse primer, 17 µl of Emerald master mix, and nuclease-free water to make up the final volume. The amplification program consisted of an initial denaturation at 95°C for 5 min, followed by 38 cycles of denaturation at 94°C for 3s, primer annealing at 60°C for 1 min, extension at 72°C for 1 min and a final extension at 72°C for 7 min. PCR products were resolved on 1% agarose gel containing ethidium bromide (0.2 mg/ml) for 40 min at 80 volts using a horizontal gel electrophoresis unit. TBE buffer (90 mM Tris-borate and 2 mM EDTA) was used for gel preparation and as gel running buffer. After electrophoresis, amplification was observed in the Alpha Imager Imaging system (Alpha Innotech). The PCR amplified products for CD MYB59 were sequenced at Delhi University, South Campus, New Delhi.

#### Bioinformatics analysis.

Nucleotide and amino acid sequences were analysed using the NCBI and EXPASY tools. Amino acid sequence alignment was performed with the Clustal X program (Larkin et al. 2007) [48], while BLAST searches were carried out using the National Centre for Biotechnology Information website. Phylogenetic analysis was carried out in MEGA 12.0 [49]. The motif identification for these genes was detected using two independent programs, namely, PROSITE [50] and CDD [51]. The tertiary structures of *MYB59* were predicted using Phyre2 software [52]. The Jmol [53] program was used for the graphical representation of tertiary protein structure.

## Results

### Genetic variation analysis of sugarcane genotypes during drought

Eighteen genotypes (ISH-4, Co canes-14) were evaluated for nine morpho-physiological and two biochemical traits at the formative growth phase for drought stress tolerance. Drought stress was imposed by withholding irrigation for 30 days from 90 to 120 DAP. The results showed a significant variation (P ≤ 0.05 or P ≤ 0.01) among the genotypes for all traits measured under drought stress. ANOVA between genotypes (G) and water regimes (control and drought stress) revealed individual and interactive effects and are presented in Tables 1 and 2.

**Table 1:** Analysis of variance for morpho-physiological traits in sugarcane at 120 DAP.

Source	df	TC	CT^F^ (cm)	No. of IN	CH^F^ (cm)	SPAD	LAI (m^2^m^-2^)	RWC (%)	Canopy temperature (°C)	*Fv/Fm*	NRase activity (μmole NO_2_ g^-1^ FW)	Proline (μmole g^-1^ FW)
**Genotype (G)**	17	1165.57^**^	0.38^**^	17.45^**^	1049.09^**^	64.79^**^	3.32^**^	52.63^**^	1.80^*^	0.02^*^	0.35^**^	49.07^**^
**Treatment (T)**	1	5166.86^**^	1.31^**^	211.84^**^	21132.25^**^	4251.03^**^	101.84^**^	6370.94^**^	292.82^*^	0.64^**^	15.15^**^	1584.75^**^
**Genotype x treatment (G x T)**	17	42.05^**^	0.01^**^	0.33^**^	42.59^**^	3.65^**^	0.14^**^	14.95^**^	1.09^ns^	0.02^**^	0.01^**^	10.89^**^

TC–Tiller Count; CTF–Cane Thickness Formative phase; No. of IN–Number of Internodes; CHF–Cane HeightFormative phase; SPAD–Soil Plant Analysis Development; LAI–Leaf Area Index; RWC–Relative Water Content; Fv/Fm–Variable Fluorescence/Maximum Fluorescence and NRase–Nitrate Reductase. ^**, *,^ and ns indicates significance at *P* ≤ 0.01, *P* ≤ 0.05 and non-significant respectively.

**Table 2:** Analysis of variance for cane yield parameters and quality traits in sugarcane at 300 DAP.

Source	df	NMC (t/ha)	CT^M^ (cm)	CH^M^ (cm)	SCW (kg)	BRX%	Sucrose%	CCS%	CCSY (t/ha)	Cane yield (t/ha)
**Genotype**	17	1099.08**	0.31**	3560.53**	0.10**	19.58**	18.33**	8.48**	5.79**	600.56**
**Treatment**	1	4966.72**	2.96**	49612.50**	1.51**	76.96**	68.66*	32.21**	169.46**	7494.34**
**Genotype x Treatment**	17	22.91 **	0.03**	222.12**	0.01*	0.07**	0.08^ns^	0.35**	0.30**	7.84**

NMCM–Number of Millable Canes Maturity phase, CTM–Cane Thickness Maturity phase, CHM–Cane Height Maturity phase, SCW–Single Cane Weight, CCS–Commercial Cane Sugar; CCSY–Commercial Cane Sugar Yield. ^**, *,^ and ns indicates significance at *P* ≤ 0.01, *P* ≤ 0.05 and non-significant respectively.

**Fig 1:**
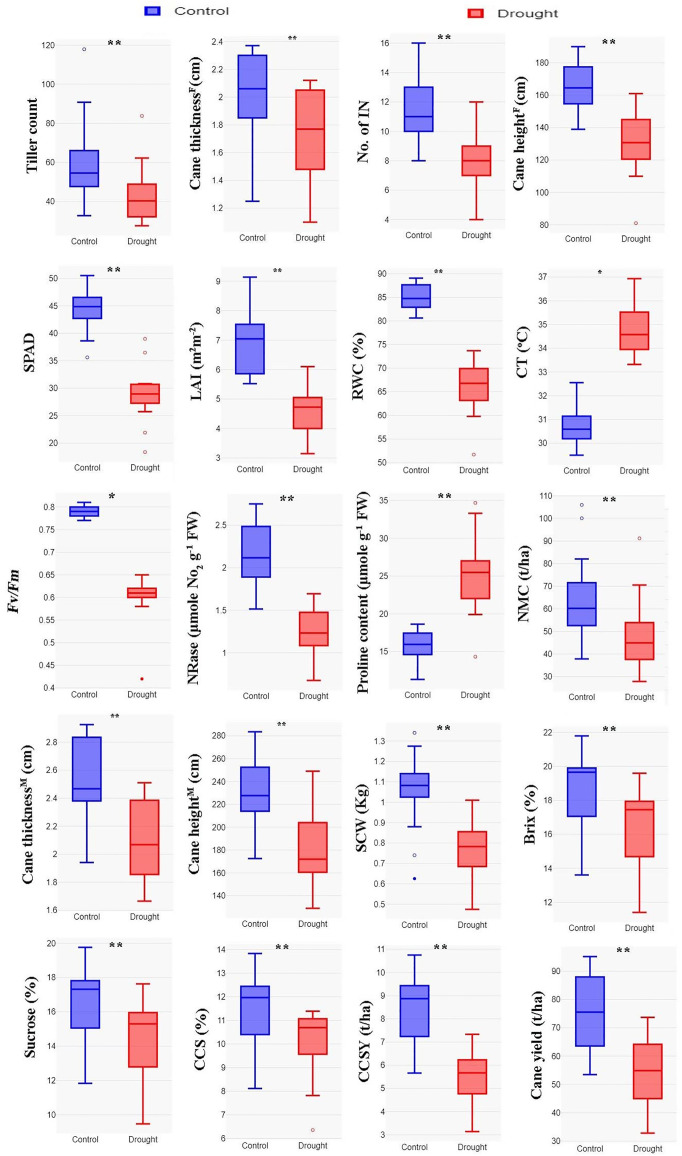
Box plots explaining descriptive statistics of drought stress effect on morph-physiological and biochemical traits at the formative and maturity growth phase. TC-tiller count; CT^F^-cane thickness^formative phase^ (cm); No. of NI-number of internodes; CH^F^-cane height^formative phase^ (cm); SPAD–soil plant analysis development; LAI-leaf area index (m^2^m^-2^); RWC-relative water content (%);canopy temperature (%); Fv/Fm–variable fluorescence/maximum fluorescence; NRase-nitrate reductase (μmole NO_2_ g^-1^ FW); PRO-proline content (μmole g^-1^ FW); NMC-number of millable canes(t/ha); CT^M^–cane thickness^maturity phase^(cm); CH^M^ –cane height^maturity phase^(cm); SCW–single cane weight(Kg); BRX–brix (%); SUC-sucrose(%); CCS-commercial cane sugar (%); CCSY-commercial cane sugar yield (t/ha) and CY-cane yield (t/ha).

The mean performance of genotypes for the traits studied during control and drought stress at the formative growth phase (120 DAP) are presented in S4 and S5 Tables, respectively. The box plots representing descriptive statistics are given in Fig 1. Among the morphological parameters, the tiller count (TC), cane thickness^F^ (cm), and cane height^F^ (cm) showed downturn performance during drought with an overall decrease of 27.98%, 13.48%, and 21.19%, respectively. The effect of drought stress had also caused substantial variations in individual genotypes, explaining the genetic variations present between and among the interspecific hybrids as well as the Co canes. Among the genotypes, AS-04-1687 registered the highest TC (94.80%) improvement over the mean performance of the trait during drought stress. It also recorded the lowest reduction for the number of internodes (24.59%), cane height^F^ (15.26%,) and the least reduction for cane thickness^F^. The genotype Co 775 registered the highest reduction for all the above morphological traits studied.

Similarly, the mean performance of genotypes for the above traits was also recorded at the maturity growth phase (300 DAP), i.e., after drought stress recovery, and are presented in S6 and S7 Tables, respectively, and an aerial view of the maturity growth phase captured by drone owned by our institute, Indian Council of Agriculture Research – Sugarcane Breeding Institute is given in S1 Fig. Mean performance for yield traits *viz*., NMC, cane thickness^M^, cane height^M^ and SCW, were reduced by 26.04%, 16.16%, 22.90% and 27.29%, respectively, during drought. Juice quality was reduced by 12.05%, and overall CCS and cane yield were reduced by 35.82% and 27.24%, respectively. The genotype AS-04-1687 registered the lowest reduction of 13.95% and 12.17% for NMC and cane height, respectively, indicating the faster recovery of drought stress. Comparatively, all ISH clones performed better for NMC (69.07 t/ha) and cane height (223.25 cm) than Co canes. The trait SCW was largely reduced by drought stress, with an overall mean of 0.77 kg, over 1.06 kg during control. Mean cane yield during drought was higher in ISH clones (68.16 t/ha) than Co canes (51.80 t/ha).

### Physiological response of sugarcane genotypes under stress

The effects of drought on the chlorophyll content of sugarcane genotypes were assessed during the drought period. The results showed a significant variation among the genotypes for all traits measured except the interactive effect (G × T) of canopy temperature under drought stress (Table 1). The mean performances of all genotypes decreased during drought stress for all traits except for proline content and canopy temperature. Among the traits studied, NRase activity showed the highest reduction of 42.94% under drought stress, followed by SPAD (35.04%), LAI (34.11%) and No. of IN (31.21%). RWC and *Fv/Fm* also showed downturn performance during drought with an overall decrease of 22.20% and 24.14%, respectively. Mean proline content and canopy temperature increased by 58.03% and 13.18%, respectively, during drought stress. The highest SPAD value of 38.99 and a large LAI of 6.10 (m^2^m^-2^) were observed in AS-04-1687 (above 30.00). Mean RWC of ISH ranged from 66.59 to 73.69%, whereas in Co canes it was 51.72 to 70.93% under drought stress. *Fv/Fm* ranged from 0.77 to 0.81 in control, while it ranged from 0.42 to 0.65 under drought stress. Mean NRase activity and proline under control were 2.16 and 15.80, respectively, and while at drought stress, it varied from 1.24 and 25.18, respectively. Overall mean canopy temperature was increased by 4°C under drought stress over the control.

### Multivariate evaluation of sugarcane genotypes

#### Correlation analysis.

**Fig 2:**
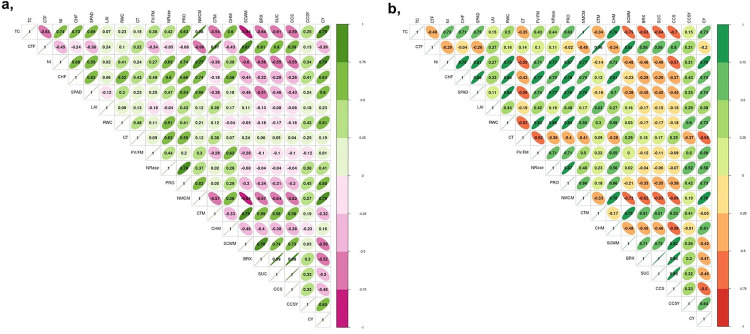
Correlogram of 20 traits studied in 18 sugarcane genotypes. (a) Control and (b) Drought stress. Higher colour intensity illustrates higher correlation. TC-tiller count; CT^F^-cane thickness^formative phase^ (cm); No. of NI-number of internodes; CH^F^-cane height^formative phase^ (cm); SPAD–soil plant analysis development; LAI-leaf area index (m^2^m^-2^); RWC-relative water content (%); canopy temperature (%); Fv/Fm–variable fluorescence/maximum fluorescence; NRase-nitrate reductase (μmole NO_2_ g^-1^ FW); PRO-proline content (μmole g^-1^ FW); NMC-number of millable canes(t/ha); CT^M^–cane thickness^maturity phase^(cm); CH^M^ –cane height^maturity phase^(cm); SCW–single cane weight (Kg); BRX–brix (%); SUC-sucrose(%); CCS-commercial cane sugar (%); CCSY-commercial cane sugar yield (t/ha) and CY-cane yield (t/ha).

Pearson’s correlation coefficients for the 20 traits studied under control and drought stress are given in Fig 2a and 2b. During drought, most of the yield traits had positive and significant associations with cane yield (t/ha), but correlation coefficients largely increased during drought in comparison to the control. Under drought stress, all morphological traits showed a significant positive correlation with cane yield (t/ha) except cane thickness^F&M^, canopy temperature, SCW, brix%, pol% and CCS%.

#### Principal component analysis (PCA).

**Fig 3:**
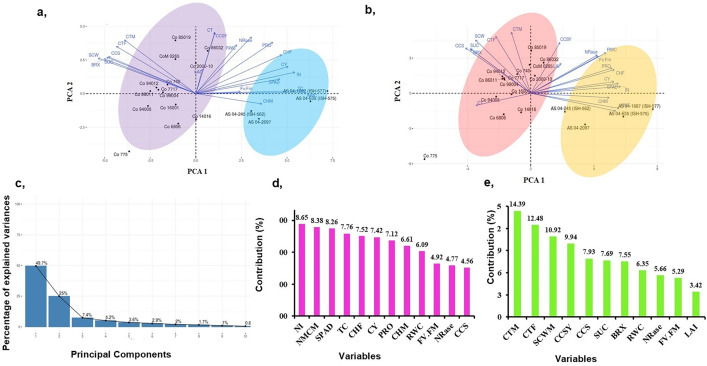
Principal component analysis (PCA) of 18 sugarcane genotypes based on variability in morpho-physiological and biochemical traits. (a) Control, (b) Drought stress, (c) Scree plot, (d) Contribution of variables to PC1 and **(e)** Contribution of variables to PC2. TC-tiller count; CT^F^ -cane thickness^formative phase^ (cm); No. of NI-number of internodes; CH^F^-cane height^formative phase^ (cm); SPAD–soil plant analysis development; LAI-leaf area index (m^2^m^-2^); RWC-relative water content (%); canopy temperature (%); Fv/Fm–variable fluorescence/maximum fluorescence; NRase-nitrate reductase (μmole NO_2_ g^-1^ FW); PRO-proline content (μmole g^-1^ FW); NMC-number of millable cane(t/ha); CT^M^–cane thickness^maturity phase^(cm); CH^M^–cane height^maturity phase^(cm); SCW–single cane weight (Kg); BRX–brix (%); SUC-sucrose(%); CCS-commercial cane sugar (%); CCSY-commercial cane sugar yield (t/ha) and CY-cane yield (t/ha).

Principal component analysis (PCA) was done for 20 traits evaluated in 18 genotypes at control (C) and drought stress (D) treatments to study the diversity of genotypes and the association of traits. PCA-biplots of C and D and the scree plot are given in Fig 3a–3c. In control, PC_1_ and PC_2_ explained 44.1% and 20.1% of total variability, which was contributed by NMC, TC, No. of IN, SCW, and cane thickness^F^, CCSY, NRase, and cane thickness^M,^ respectively. In drought stress, No. of IN, NMC, SPAD, TC, CH^F^, proline, and RWC contributed largely to PC_1,_ explaining 49.70% of total variability (Fig 3d) and the remaining 25% of variability was explained by PC_2_ which was defined by CT^F^, CT^M^, SCW, and CCSY (Fig 3e). In both control and drought, PCA analysis identified two groups of traits and two clusters of genotypes (Fig 3a and 3b).

#### Hierarchical cluster analysis.

**Fig 4:**
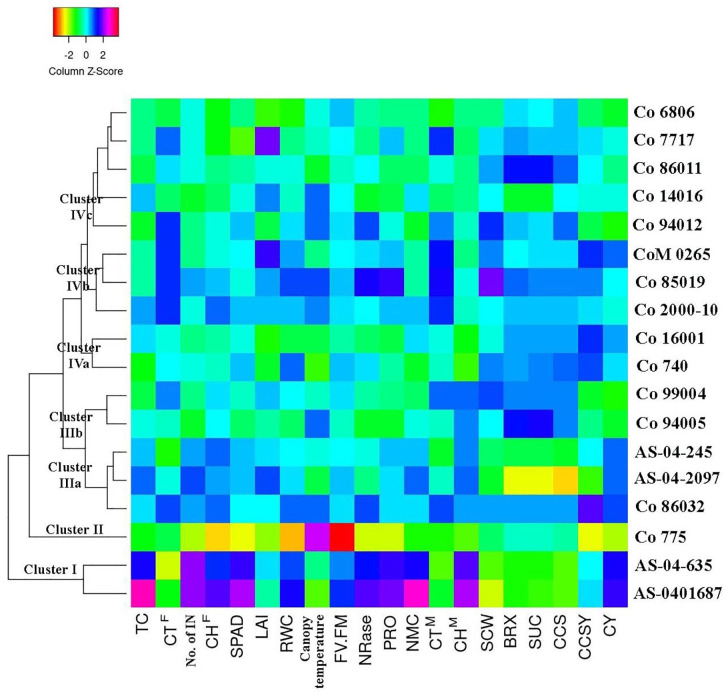
Hierarchical clustering and heatmap analysis of 18 sugarcane genotypes under drought stress condition. TC-tiller count; CT^F^-cane thickness^formative phase^ (cm); No. of NI-number of internodes; CH^F^-cane height^formative phase^ (cm); SPAD–soil plant analysis development; LAI-leaf area index (m^2^m^-2^); RWC-relative water content (%);canopy temperature (%); Fv/Fm–variable fluorescence/maximum fluorescence; NRase-nitrate reductase (μmole NO_2_ g^-1^ FW); PRO-proline content (μmole g^-1^ FW); NMC-number of millable cane(t/ha); CT^M^–cane thickness^maturity phase^(cm); CH^M^–cane height^maturity phase^(cm); SCW–single cane weight(Kg); BRX–brix (%); SUC-sucrose(%); CCS-commercial cane sugar (%); CCSY-commercial cane sugar yield (t/ha) and CY-cane yield (t/ha).

Hierarchical cluster analysis grouping the 18 clones with 20 traits studied under drought is shown in Fig 4. The result revealed four major clusters, such as clusters I, II, III and IV and clusters III and IV were further subdivided as IIIa, IIIb, IVa, IVb and IVc (Fig 4). Among the clusters, genotypes belonging to cluster I had the highest NMC, cane height and cane yield and also performed best for all the physiological and biochemical traits studied compared with all other clusters. Co 86032, identified as drought stress tolerant, had a high cane yield and also, with on par performance to ISH clones for most physio-biochemical traits, was grouped with ISH clones in cluster IIIa. Genotypes with the highest brix and sucrose % were grouped in cluster IIIb. Cluster IVa (cane thickness, SCW) and IVb (CCS yield) were recorded with the highest mean for yield traits.

### Expression profiling of drought stress-induced genes/transcription factors

Based on the morpho-physiological and biochemical performance, four interspecific genotypes (AS-04-1687, AS-04-635, AS-04-2097, and AS-04-245), along with a tolerant (Co 85019) and susceptible (Co 775) genotype, were chosen for expression profiling. The responses of the ten drought stress-induced genes were evaluated across these genotypes. Among the six sugarcane genotypes studied, AS-04-1687 during drought stress exhibited the highest expression of NAC with a 9.36-fold increase over the control, followed by AS-04-635 with a fold increase of 7.64. While the tolerant and susceptible varieties exhibited a fold change of 3.26 and 1.12 for Co 85019 and Co 775, respectively. The expression of MYB59 was highest in AS-04-1687, followed by AS-04-635, AS-04-2097 and AS-04-245, with a fold increase of 19.36, 15.42, 11.29 and 9.94, respectively. The tolerant (Co 85019) and the susceptible genotype (Co 775) had a fold increase of 14.88 and 1.14, respectively, for MYB59. It was observed that from this study, the expression of POD was high in AS-04-1687 with a fold increase of 13.21 over the control, followed by AS-04-635 with a fold increase of 9.64. The fold increase of POD expression in AS-04-2097 and Co 85019 was 4.98 and 4.37, respectively, whereas the least fold increase of 1.23 was observed in Co 775.

**Fig 5:**
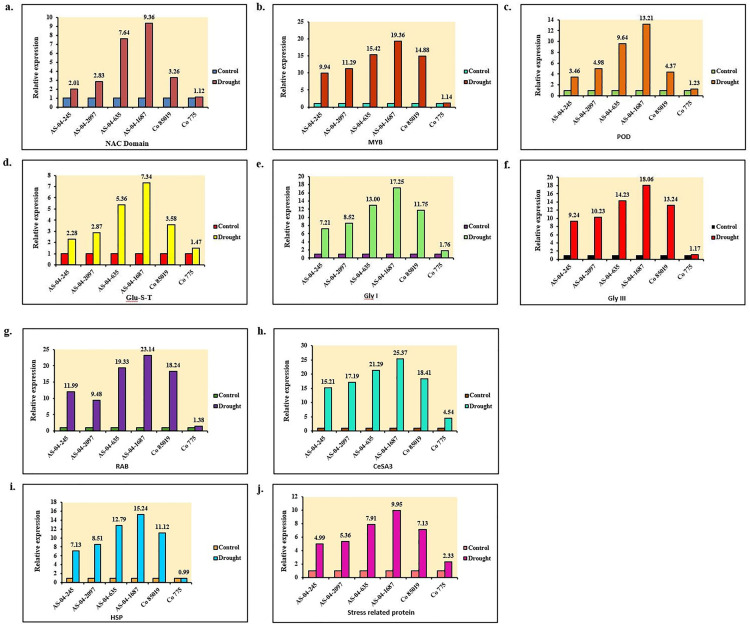
Expression analysis of drought responsive genes. (a) NAC domain (N acetyl cysteine), (b) MYB (Myeloblastosis), (c) POD (Peroxidase), (d) Glu-S-T (Glutathione S Transferase), (e) Gly I (Glyoxylase I) and (f) Gly III (Glyoxylase III) during the formative growth phase in the stem of sugarcane genotypes. b. Effect of drought stress on gene expression. (g) RAB (Responsive to Abscisic acid), (h) CesA 3 (Cellulose synthase), (i) HSP (Heat shock protein), and (j) Stress-related protein during the formative growth phase in the stem of sugarcane genotypes.

**Fig 6:**
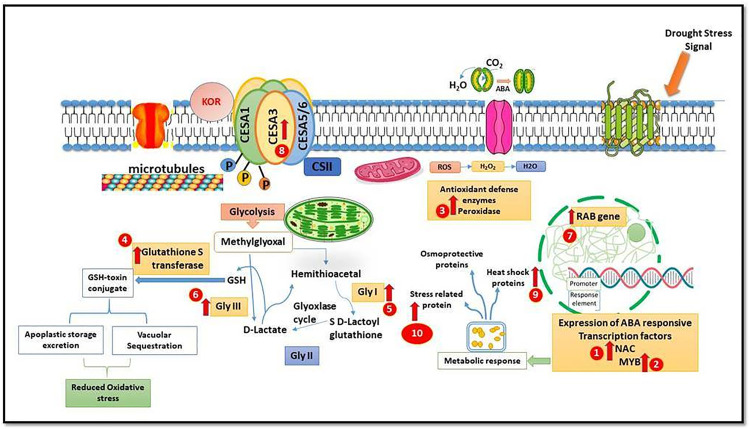
Regulation of drought stress stress signalling pathway. 1. N-acetyl cysteine (NAC), 2. Myeloblastosis – (MYB), 3. Peroxidase (POD), 4. Glutathione S transferase (GST), 5. Glyoxylase I (GLY **I)**, 6. Glyoxylase III (GLY **III)**, 7. Responsive to Abscisic acid (RAB), 8. CesA3 – Cellulose synthase, 9. Heat shock proteins (HSP) and 10. Stress-related proteins.

GST mitigates oxidative stress caused by ROS, thereby preventing cellular damage in plants. Among the four interspecific genotypes, AS-04-1687 had the highest fold increase of 7.34 over the control during drought stress, whereas the least fold increase of 2.28 was observed in AS-04-245. The tolerant and susceptible genotypes had a fold increase of 3.58 and 1.47, respectively, for GST. Two GLY genes, *viz*., GLY I and GLY III, revealed that the expression levels of GLY III were higher in all the genotypes, including the tolerant and susceptible ones, than GLY I. GLY I ranged from a fold increase of 17.25 in AS-04-1687 to 7.21 in AS-04-245, while GLY III ranged from 18.06 in AS-04-1687 to 9.24 in AS-04-245. The expression in the tolerant genotype was higher in drought stress than in the control in both GLY I and GLY III. The RAB gene plays an important role in various physiological functions of a plant cell, enabling it to withstand several environmental stresses. In this study, like all other genes, the highest expression of RAB under drought stress witnessed a fold increase of 23.14, 19.33, 9.48 and 11.99 in AS-04-1687, AS-04-635, AS-04-2097 and AS-04-245, respectively. The tolerant genotype exhibited a fold increase of 18.24 for RAB, whereas in the susceptible genotype (Co 775) it was 1.38. Cellulose synthase genes play a key role in the synthesis of cell wall components in plants. Unlike other genes, the expression of CesA3 was exorbitantly high in all genotypes with a fold increase of 25.37, 21.29, 17.19 and 15.21 in AS-04-1687, AS-04-635, AS-04-2097 and AS-04-245, respectively. Regarding the expression of heat shock proteins, the highest response was observed in AS-04-0687 with a fold increase of 15.24, followed by AS-04-635, AS-04-2097 and AS-04-245 with fold increases of 12.79, 8.51 and 7.13, respectively. Upregulation of heat shock proteins was also observed in the control genotype Co 85019. Plants exhibit a variety of stress-related proteins in response to drought stress, primarily to stabilize their cellular components. In our investigation, among the interspecific hybrids, the expression of LEA proteins was found to be highest in AS-04-1687, followed by AS-04-635, AS-04-2097 and AS-04-245, with a fold increase of 9.95, 7.91, 5.36 and 4.99, respectively. A fold increase of 7.13 was observed in the tolerant genotype and 2.33 in the susceptible one (Fig 5a and 5b). A schematic diagram showing the networking of genes and their regulations of the stress signalling pathway during drought stress is furnished in Fig 6.

### Identification and cloning of the drought stress-induced transcription factor MYB59

**Fig 7:**
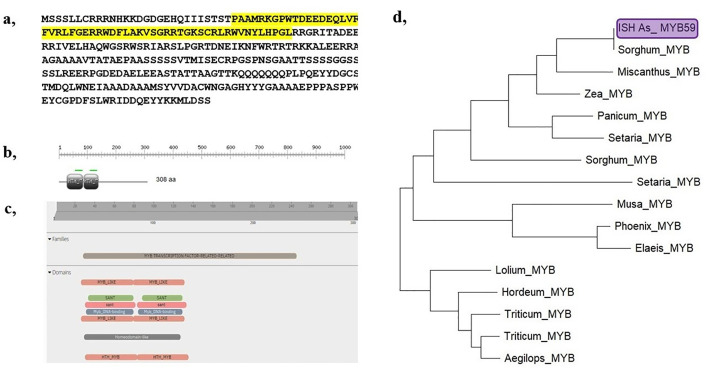
Domain analysis and phylogenetic relationship of the transcription factor Myb59. (a) Translated amino acid sequence of the isolated transcription factor, *Myb59*, **(b)** Domain identification of the sequenced transcription factor, *Myb59* and, **(c)** Depicting domains related to *Myb* transcription factor, **(d)** Phylogenetic relationship of the coding sequence (CD) of MYB59.

From the expression profiling data, the MYB59 transcription factor was identified and selected for cloning and characterisation from the drought-tolerant interspecific hybrid AS-04-1687. The PCR amplified product showed a fragment size of approximately 900 bp (S2 Fig). The amplified product was gel eluted, ligated with pJET1.2 and transformed into *E. coli* Dh5α. Nearly 20 colonies were chosen for the cloning confirmation by colony PCR used for screening of positive colonies (S2 Fig). Plasmid DNA was isolated from the positive colonies and reconfirmed through PCR using plasmid DNA as a template (S2 Fig). The positive colonies were sequenced (S2 Fig) and the sequence analysis showed that MYB59 has an open reading frame (ORF) of 927 bp that encodes 308 amino acids (Fig 7a). The predicted molecular weight of the deduced protein was 34.248 Da with an isoelectric point (p*I*) of 6.97 calculated by the ExPASy Proteomic Server. Sequence analysis of MYB 59 revealed two helix-turn-helix (HTH) domains present at positions 29–85 and 86–136 (Fig 7b), indicating that it belongs to the R2R3 gene family of MYB. This repeat region is involved in DNA-binding, where R2 and R3 bind directly to the DNA major groove. The InterPro scan resulted in several domains belonging to the MYB transcription factor-related family, which are given in Fig 7c. A phylogeny tree was constructed, and it was observed that the identified transcription factor MYB59 in sugarcane ISH clone AS-04-1687 showed close similarity with *Sorghum,* followed by *Miscanthus* and *Zea mays* transcription factor MYB59-like (Fig 7d).

## Discussion

### Morphological response of sugarcane interspecific hybrids to drought stress

Drought stress imposed during the formative phase resulted in major changes in morphological traits. This study also showed that the growth phase, the level of stress, and genetic variations had a significant impact on the tolerance responses. All the genotypes showed an overall decrease in the morphological traits, *viz*., tiller count, cane thickness and cane height during drought. Earlier reports [54–56] also reported a reduction in leaves, tillers, roots and their total dry weights during drought, which was similar to our results. An investigation carried out by Zhao et al. (2010) [56] and Endres et al. (2018) [57] reported that the genotype producing many tillers will not always have a higher yield because water stress may also result in the production of high tillers but smaller stalks, thereby affecting cane yield. In concurrence with the above finding, the present study identified the agronomically desirable combination of higher tiller count with taller canes during drought stress in ISH clones.

### Physiological responses of sugarcane interspecific hybrids to drought stress

Leaves are the primary site of photosynthesis and transpiration, which exhibit structural modifications to adapt to adverse environmental conditions. Under drought stress, Misra et al. (2020) [6] observed a 31.11% reduction in leaf width, and Endres et al. (2018) [57] recorded a 29.7% reduction in leaf length. In the present study, LAI was reduced over the control during drought stress. It was reported that reduced leaf width and maintenance of high leaf area under water stress were important indicators for tolerance and productive genotypes [58,59]. The highest reduction of LAI in Co 775 and the lowest in AS-04-1687 and Co 85019 delimited the stress response between tolerance and susceptible genotypes. All the genotypes studied reported decreased SPAD, RWC and *Fv/Fm* and increased canopy temperature during drought stress, which was similar to the earlier reports. Jangpromma et al. (2010) [11] also reported that early-season drought stress reduces the SPAD index in sugarcane. Earlier, Hemaprabha et al. (2008 & 2013) [60,61] also identified Co 85019, Co 740 and Co 86032 as abiotic stress tolerance varieties. The clone Co 775 registered the lowest values for all the morphological and physiological traits, indicating that it was less susceptible to drought stress. The above finding was substantiated by the study of Hemaprabha et al. (2013) [61], where Co 775 was reported as drought stress-susceptible.

### Biochemical responses under drought stress

An increase in proline content of 77.3% was reported by Li et al. (2021) [19] under drought stress, which was in accordance with our results, thereby linking the accumulation of proline and drought stress response. Mean proline content (antioxidant-compatible osmolyte) was increased, and NRase was reduced during drought stress. Garkar et al. (2011) [62] recorded a 41.31% decrease in NRase activity under water stress and evidenced that tolerant varieties, *viz*., Co 86032 and CoM 0265, had high activity. The present study observed the highest individual proline increase (87.08%) and the lowest NRase reduction (38.36%) in AS-04-1687 (ISH), which also outperformed the other tolerant Co canes for most morpho-physiological traits studied. Hence, AS-04-1687 can also be used as the best candidate for early drought stress tolerance assessment in sugarcane.

### Multivariate assessment of sugarcane under drought-stressed conditions

Pearson’s correlation test revealed that most of the studied morpho-physiological and biochemical traits had significantly more positive correlation with cane yield under drought stress than in the control. Khaled et al. (2018) [59] explained that plant height was positively correlated with cane yield, and irrigation was necessary for cane elongation; hence, drought stress will result in reduced cane height. According to Natarajan et al. (2020) [63], Mahadevaiah et al. (2021) [64] and Arun Kumar et al. (2020) [65], among the yield components, cane height was the highest positively correlated trait to cane yield under drought stress, and this was also consistent with our study, where cane height^F^ has the highest significant positive correlation to cane yield (r = 0.74**) at D. High relative water content (RWC) under drought stress aids in the maintenance of normal metabolic activities within a cellular system (Huang et al. 2020). Wirojsirasak et al. (2024) [20] studied a significant positive correlation of RWC to cane yield under drought stress in sugarcane. Similar to the above-mentioned reports, the present study also found that SPAD and RWC were the highest positively correlated physiological traits to cane yield, with r = 0.74** and r = 0.73**, respectively. Studies by Garkar et al. (2011) [62]; Gomathi et al. (2011) [32]; Kumar et al. (2023); and Wirojsirasak et al. (2024) [20] also reported on the positive correlation of cane yield with NRase activity, LAI, SPAD, RWC, *Fv/Fm* and CCSY under drought stress in sugarcane. Drought stress in the present study stimulated high proline accumulation with high positive correlation coefficients (r = 0.74**) to cane yield, and hence can be used as a biomarker to screen adaptive response for stress. Similar reports on high proline synthesis under drought stress were also studied by Solanki and Sarangi (2014) [66], Abdelghany et al. (2021) [67], and Akter et al. (2023) [68].

Principal component analysis (PCA), a multivariate analysis, was utilized in the present study to identify the primary selection character for screening drought stress tolerance. The PCA revealed that traits *viz.*, No. of IN, NMC, SPAD, TC, CH^F^, proline, RWC, CT^F^, CT^M^, SCW and CCSY as important traits in characterizing variations in sugarcane genotypes under drought stress. Ashgan A et al. (2023) [69] reported that proline, chlorophyll, RWC and brix% highly influence variation in sugarcane under drought stress. In the present study, PCA clustered ISH clones and Co canes into two distinct clusters, explaining the genetic variation present in the interspecific hybrid and commercial hybrid studied.

Hierarchical clustering analysis provides clear insight into inter-relationships between genotypes and traits under stress [20,70]. In the present study, each cluster was grouped with genotypes exhibiting varied degrees of tolerance response. Cluster analysis evidenced differences in tolerance response by separately grouping genotypes *viz*., AS-04-1687, AS-04-635, Co 86032, Co 85019, CoM 0265, AS-04-2097, AS-04-245 and Co 2000−10 demonstrated high tolerance ability as evaluated by all 20 traits in clusters I, IIIa and IVb and whereas the susceptible genotype Co 775 [60] was solely clustered in cluster II. Genotypes in clusters IIIb and IVa had the highest mean for juice parameters (brix%, sucrose%) and CCSY under D than CT and hence established their tolerance response as indicated by increased juice quality. Genotypes grouped in cluster IVc had moderate to susceptible tolerance with a mean cane yield of 48.8 t/ha, near to the cane yield of the susceptible variety Co 775. Drought-susceptible variety Co 775 was separately clustered in PCA and hierarchical cluster analysis under drought stress. Genotypes *viz*., AS-04-1687, AS-04-635, AS-04-245, AS-04-2097, Co 86032, Co 85019, CoM 0265, Co 2000−10, Co 94012 and Co 740 with high to moderate adaptation to drought stress were also clustered accordingly to their varied tolerance both in PCA and hierarchical cluster analysis.

### Drought stress-specific gene expression in sugarcane

Expression studies on the four interspecific hybrids and Co 85019 provided valuable insights into the drought tolerance mechanisms in sugarcane. These genotypes were selected based on the previous reports which demonstrated their tolerance to various abiotic stresses such as drought, salinity and winter [64,71,72]. NAC, the largest transcription factor gene family in plants and the precursor of the amino acid cysteine, plays a pivotal role during heat, cold and drought tolerance. In this study, we have recorded an increased expression of the NAC transcription factor during drought stress. Our results were in accordance with the earlier reports by [73], in which they reported that the expression of NAC6 was upregulated 30 times in drought-stressed conditions over the well-watered sugarcane plants. Similarly, another report by [74] showed that the upregulation of NAC in a sugarcane variety, Co 94008, was observed during drought stress. They also reported that the NAC transcription factor, in addition to scavenging ROS, plays a vital role in the synthesis of chaperones, reducing spikelet sterility and improving stress tolerance in transgenic rice expressing *EcNAC67* [75].

Transcription factor MYB59 plays essential roles in drought tolerance by regulating the expression of genes involved in nutrient uptake and transport. It was found that a plethora of developmental processes were regulated by the MYB59 transcription factor, which mainly included light-dependent signalling pathways [76], tolerance to biotic and abiotic stresses [77,78], biosynthesis of secondary metabolites [79], etc. Our study has shown that the sugarcane interspecific hybrids (AS-04-1687, AS-04-635, AS-04-2097 and AS-04-24) had recorded the highest expression of MYB transcription factor, indicating their tolerance potential. In a study of the expression profile of the alternatively spliced transcript of sugarcane, MYB was higher in PEG-induced drought stress conditions [80]. In our investigation, higher levels of proline were detected, which might be due to the upregulation of MYB59 in drought stress-tolerant genotypes. Our results paralleled the findings of [81], where overexpression of a sugarcane-derived transcription factor, SoMYB18, in transgenic tobacco resulted in a higher accumulation of proline, contributing to improved drought stress tolerance. Furthermore, upregulation of transcription factors such as MYB and NAC was associated with the drought tolerance mechanism in sugarcane with a similar stress duration of 30 days, was also reported [82].

ROS scavenging enzymes, such as peroxidases, play a crucial role in plant growth and development, particularly in eliminating hydrogen peroxide [83]. Our findings of increased peroxidase expression in tolerant genotypes align with studies in tobacco [84] and soybean [85]. These studies demonstrated that drought stress tolerance was linked to heightened expression of peroxidase genes like CrPRX01 and GmPOD40. Significant upregulation of GST was observed in drought-tolerant sugarcane progenies from a mapping population derived from Co 740 (drought-tolerant) and Co 775 (drought-sensitive) [86], which matches our experimental results. Additionally, GST was uniquely detected in the drought-tolerant sugarcane variety SP81–3250 during water scarcity [87]. Methyl glyoxal, a byproduct of glycolysis, increases during drought conditions [88]. Upregulation of glyoxalase genes may facilitate methylglyoxal detoxification, helping to maintain cellular homeostasis. Elevated expression levels of glyoxalase genes were reported in studies involving *Erianthus arundinaceus* and a commercial sugarcane hybrid [89]. Furthermore, increased expression of glyoxalase genes in Co 86032, a promising sugarcane hybrid, coincided with higher chlorophyll and proline content [90], consistent with our findings where we recorded increased expression of GST, Gly I & III in AS-04-1687, contributing to drought tolerance.

Under any abiotic stress, the cell wall is the first component to receive the stimuli and alert the plant system. Cellulose synthase (CesA) genes and cellulose synthase-like genes responsible for the synthesis of cellulose and hemicellulose, respectively, were either upregulated or suppressed during drought stress conditions in varied crops [4,91,92]. In our present investigation, the *CesA*3 gene had relatively elevated levels of gene expression, which marked the evidence of cell wall strengthening in all the genotypes except the susceptible one. Parallel to our findings, the expression of HSP 70 was hugely upregulated in a drought stress-tolerant sugarcane cultivar RB867515 [93], under drought-stressed circumstances. Earlier, Augustine et al. 2015 [30] reported a fold increase greater than 2000 in the gene expression of EaHsp70 obtained from *E. arundinaceous* in drought stress conditions. In our study, the genotype AS-04-1687 had the highest upregulation, indicating that the genotype has better drought stress tolerance, possibly due to the pivotal role portrayed by the heat shock proteins in maintaining cellular homeostasis.

### Characterisation of drought-induced MYB transcription factor

MYB TFs are characterized by the presence of the MYB domain involved in DNA binding and are classified based on the number of repeats present in their sequences, varying from one to four. Each repeat consists of 52 amino acids which form three α-helices, where the second and the third α-helices are involved in the formation of a helix–turn–helix (HTH) fold [94]. In plants, the majority of MYB TFs contain two repeats, and they belong to the R2R3-MYB subfamily. [95] Many researchers have identified more than 100 MYB TFs from different species. Based on the conservation of the DNA-binding domain and amino acid motifs in the C-terminal domains, they were subdivided into 23 subgroups. Among them, the R2R3-MYB TFs play central roles in the control of plant-specific processes, which include primary and secondary metabolism, developmental processes, and response to abiotic and biotic stresses [96]. MYB59 transcription factors have been proven to enhance the tolerance to drought stress in several crops [97–99]. It is also involved in plant growth and stress responses, especially during drought and salinity stress, by acting as a negative regulator of Ca signalling and homeostasis. Hence, the identified and cloned MYB59 transcription factor might be used for transgenic development for drought stress/salinity stress tolerance.

## Conclusion

Screening of eighteen sugarcane genotypes for drought stress tolerance witnessed remarkable differences for all traits except for proline content and canopy temperature. Overall, drought stress impacted most of the traits under study, and genetic variation was substantial for every trait. Correlation revealed that a higher number of internodes, cane height, NMC, SPAD, and RWC were highly correlated to cane yield under drought stress and can be utilised as an important morphological marker for drought stress tolerance. Multivariate analysis, such as PCA and cluster analysis, grouped genotypes ranging from highly tolerant to moderately tolerant, enabling the identification of tolerant genotypes suitable for use as parents in stress tolerance breeding programme.

Among the genes analysed, NAC, MYB59, RAB, CesA3 and HSP, with their elevated expression, could be possibly involved in various protective mechanisms such as oxidative stress mitigation, osmotic protection, and transcriptional regulation, thereby strongly correlating with enhanced drought tolerance. From this study, we have identified and cloned the most appropriate transcription factor, which could be taken forward for precise gene editing for drought stress tolerance. Structural characterization of the identified MYB59 transcription factor confirmed its homology with the R2R3-MYB transcription factor family, highlighting its role in transcriptional regulation under drought-stressed conditions. Furthermore, the sugarcane MYB59 exhibited the highest sequence homology with those of Sorghum and Miscanthus, indicating a conserved role in drought tolerance across related genera. This transcription factor, therefore, represents a strong candidate for marker-assisted breeding to enhance drought resilience in sugarcane. Consequently, the interspecific hybrids AS-04-1687 and AS-04-635 could be proposed as promising parental lines for drought-tolerant breeding programmes and could be further assessed for their performance in drought-prone environments.

## Supporting information

S1 Fig Aerial view of sugarcane genotypes planted in a CRBD with two replications during maturity growth phase. (a) Control and (b) Drought stress.
(TIF)

S2 Fig Molecular confirmation of MYB59. (a) PCR amplification profile of CDS of MYB59–900 bp in AS-04–1687, (b) Colony PCR confirmation of MYB59–900 bp, (c) Plasmid PCR confirmation of MYB59–900 bp and (d) Identification of the nucleotide sequence of MYB59 in sugarcane interspecific hybrid AS-04–1687.
(TIF)

S1 Table Sugarcane (*Saccharum* spp.) genotypes used in the study. (PDF)

S2 Table Details of soil nutrient. (PDF)

S3 Table Annual weather report of 2023 (January-December 2023). (PDF)

S4 Table Morpho-physiological and biochemical traits of sugarcane genotypes under controlled conditions at 120 DAP. (PDF)

S5 Table Effect of drought stress on morpho-physiological and biochemical traits at 120 DAP of sugarcane genotypes. (PDF)

S6 Table Cane yield and quality parameters of sugarcane genotypes under controlled conditions at 300 DAP. (PDF)

S7 Table Effect of drought stress on cane yield parameters and quality traits at 300 DAP of sugarcane genotypes. (PDF)
