# Supplementary figures and images for: Unravelling drought stress adaptation in sugarcane interspecific hybrids: A multi-level analysis
Source: PLoS One. 2025 Dec 12;20(12):e0338698. doi: 10.1371/journal.pone.0338698 (PMC12700406; doi:10.1371/journal.pone.0338698)

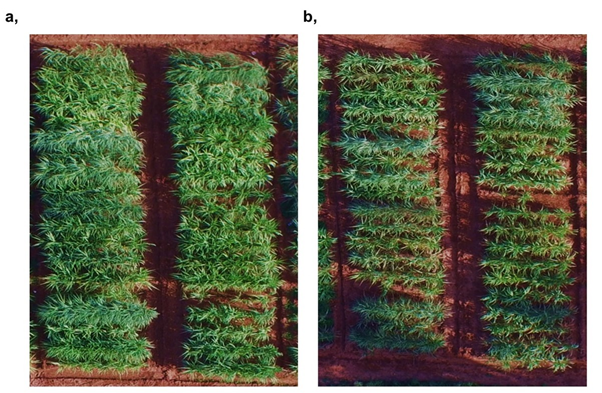

Supplement: S1 Fig — (a) Control and (b) Drought stress. (TIF) [file pone.0338698.s001.tif]

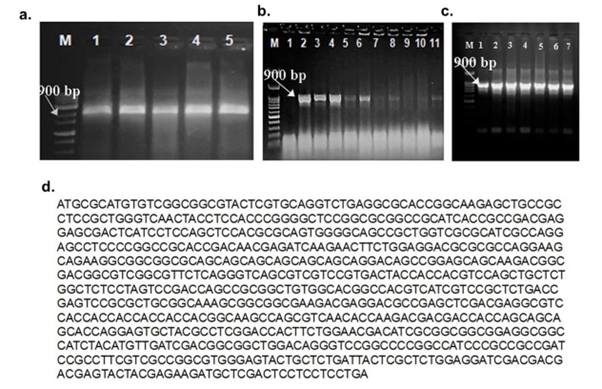

Supplement: S2 Fig — (a) PCR amplification profile of CDS of MYB59–900 bp in AS-04–1687, (b) Colony PCR confirmation of MYB59–900 bp, (c) Plasmid PCR confirmation of MYB59–900 bp and (d) Identification of the nucleotide sequence of MYB59 in sugarcane interspecific hybrid AS-04–1687. (TIF) [file pone.0338698.s002.tif]
