# Supplementary material for: Unravelling drought stress adaptation in sugarcane interspecific hybrids: A multi-level analysis
Source: PLoS One. 2025 Dec 12;20(12):e0338698. doi: 10.1371/journal.pone.0338698 (PMC12700406; doi:10.1371/journal.pone.0338698)
Supplement: S1 Table — (PDF) [file pone.0338698.s003.pdf]

S1 Table. Sugarcane (*Saccharum* spp.) genotypes used in the study.

S. No	Genotypes	Parentage	Maturity
1	AS 04-1687	BO 102 x IND 84-337	Mid-late maturing
2	AS 04-635	CoH 114 x SH 216	Mid-late maturing
3	AS 04-2097		Mid-late maturing
4	AS 04-245	Co 89029 x IND 84-394	Mid-late maturing
5	Co 740	(Co 421 x Co 440) x (Co 464 x Co 440)	Late maturing
6	Co 775	POJ 2878 x Co 371	Mid-late maturing
7	Co 7717	Co 419 x Co 775	Mid-late maturing
8	Co 6806	Co 775 x Co 798	Early maturing
9	Co 86011	Co 7314 x Co 775	Mid-late maturing
10	Co 94012	Somaclone of CoC 671	Mid-late maturing
11	Co 85019	Co 7201 x Co 775	Late maturing
12	CoM 0265	Co 87044 GC	Mid-late maturing
13	Co 14016	Co 86032 x Co 86011	Mid-late maturing
14	Co 16001	CoSnk 03-044 x Co 86002	Early maturing
15	Co 94005	Co 7201 x Co 775	Early maturing
16	Co 99004	Co 62175 x Co 86250	Mid-late maturing
17	Co 2000-10	CoC 671 x Co 86250	Mid-late maturing
18	Co 86032	Co 62198 x CoC 671	Mid-late maturing

Source: ICAR-Sugarcane Breeding Institute, Coimbatore, Tamilnadu, India.
