# Supplementary material for: Unravelling drought stress adaptation in sugarcane interspecific hybrids: A multi-level analysis
Source: PLoS One. 2025 Dec 12;20(12):e0338698. doi: 10.1371/journal.pone.0338698 (PMC12700406; doi:10.1371/journal.pone.0338698)
Supplement: S2 Table — (PDF) [file pone.0338698.s004.pdf]

S2 Table. Details of soil nutrients.

**S.No.**	**Soil composition**	**Quantity**
1	Nitrogen (kg/ha)	183.5
2	Phosphorus (P_2O_5) (kg/ha)	65.52
3	Potassium (K_2O) (kg/ha)	461.5
4	pH	8.02
5	EC (dS/m)	0.29
6	OC (%)	0.41
