# Supplementary material for: Unravelling drought stress adaptation in sugarcane interspecific hybrids: A multi-level analysis
Source: PLoS One. 2025 Dec 12;20(12):e0338698. doi: 10.1371/journal.pone.0338698 (PMC12700406; doi:10.1371/journal.pone.0338698)
Supplement: S3 Table — (PDF) [file pone.0338698.s005.pdf]

S3 Table. Annual weather report of 2023 (January-December 2023).

Month	Date	Mini Tem	RH I	RH II	Rainfall	E V P	Solar Radian
**January**	**1.1.2023**	18.6	91	33	0	5.2	414
	**2.1.2023**	20.5	84	33	0	5	442.5
	**3.1.2023**	20.5	82	39	0	5	398.3
	**4.1.2023**	20.5	83	41	0	6	380.6
	**5.1.2023**	21	84	45	0	5	354
	**6.1.2023**	20	83	43	0	5.2	389.4
	**7.1.2023**	21	80	46	0	5	354
	**8.1.2023**	21.2	89	46	0	5.6	354
	**9.1.2023**	21.5	86	45	0	5	345.2
	**10.1.2023**	16	85	39	0	5	327.5
	**11.1.2023**	16	85	32	0	6	424.8
	**12.1.2023**	16.5	84	29	0	5	433.7
	**13.1.2023**	17	83	45	0	5	424.8
	**14.1.2023**	16.5	82	31	0	4	274.4
	**15.1.2023**	15.8	95	35	0	3.8	354
	**16.1.2023**	16	82	34	0	4.2	336.3
	**17.1.2023**	20	83	38	0	5	407.1
	**18.1.2023**	16.6	88	42	0	5	362.9
	**19.1.2023**	20	83	34	0	5	424.8
	**20.1.2023**	17	82	37	0	4.6	407.1
	**21.1.2023**	18.5	81	41	0	5	415.9
	**22.1.2023**	19.7	89	41	0	5	354
	**23.1.2023**	21	82	43	0	4.4	265.5
	**24.1.2023**	22.5	86	61	0.5	5.5	380.6
	**25.1.2023**	21	82	59	0	4	177
	**26.1.2023**	20	83	41	0	4	230.1
	**27.1.2023**	18	82	30	0	4.2	345.2
	**28.1.2023**	17.5	84	23	0	4.6	380.6
	**29.1.2023**	16	87	24	0	4.4	433.7
	**30.1.2023**	21.5	82	51	0	4.6	433.7
	**31.1.2023**	22.5	81	53	0	6	300.9

Month	Date	Max	Mini	RH I	RH II	Rainfall	E V P	Solar Radian
**February**	**1.2.2023**	30.5	18.5	86	27	0	4	256.7

	**2.2.2023**	31.5	21.5	74	42	0	4.2	344
	**3.2.2023**	28.5	21.5	81	64	0	4	131.2
	**4.2.2023**	27	22	83	42	1.5	4.5	123
	**5.2.2023**	30.5	19	92	16	0	5	270.6
	**6.2.2023**	32.5	16.5	80	22	0	5.6	426.4
	**7.2.2023**	32.5	16.5	82	24	0	6	393.6
	**8.2.2023**	33.5	20.5	73	30	0	6	393.6
	**9.2.2023**	32.5	19.5	81	33	0	5	377.2
	**10.2.2023**	32	18	81	17	0	5	393.6
	**11.2.2023**	32.5	17.5	75	17	0	4.6	377.2
	**12.2.2023**	33	18	81	15	0	6	385.2
	**13.2.2023**	33	16	80	20	0	66	434.2
	**14.2.2023**	33	16	78	27	0	7	434.6
	**15.2.2023**	32.5	19	81	26	0	6	426.4
	**16.2.2023**	33.5	19.5	76	24	0	6.6	426.4
	**17.2.2023**	32.5	17.5	81	25	0	7	436.4
	**18.2.2023**	31.5	19.5	78	28	0	7	418.2
	**19.2.2023**	32.2	20	82	33	0	7	426.4
	**20.2.2023**	32	22	83	36	0	6.6	459.2
	**21.2.2023**	33	19.5	81	38	0	6	401.8
	**22.2.2023**	33.5	18	79	23	0	7	442.8
	**23.2.2023**	33.5	19	81	19	0	7	426.4
	**24.2.2023**	32.5	21.5	81	33	0	7	451
	**25.2.2023**	32.5	19.5	80	13	0	6.6	451
	**26.2.2023**	33.7	17.2	73	15	0	7.8	442.8
	**27.2.2023**	33.7	19	81	28	0	8	434.6
	**28.2.2023**	31.5	18.5	75	32	0	8	459.2

**Month**	**Date**	**Max**	**Mini**	**RH I**	**RH II**	**Rainfall**	**E V P**	**Solar Radian**

**March**	**1.3.2023**	32	23.5	80	41	0	7	377.2
	**2.3.2023**	31.5	22	86	23	0	7	246.4
	**3.3.2023**	32	15.5	72	13	0	7	438.9
	**4.3.2023**	32.5	18	81	17	0	7.4	423.5
	**5.3.2023**	33.5	22.2	56	30	0	8	423.5
	**6.3.2023**	33.5	18	74	20	0	7.6	423.5
	**7.3.2023**	33.5	20.5	85	30	0	8	446.6
	**8.3.2023**	32.5	22	84	31	0	8	385
	**9.3.2023**	32.5	21.5	76	33	0	7.6	400.4
	**10.3.2023**	32.5	22	83	23	0	7	369.6
	**11.3.2023**	33	21.5	84	25	0	7.8	400.4
	**12.3.2023**	33.4	22.8	83	27	0	8	392.7
	**13.3.2023**	33.5	22.5	75	35	0	8	354.2
	**14.3.2023**	34	21	75	32	0	8	408.1
	**15.3.2023**	35	23	83	31	0	7.6	369.6
	**16.3.2023**	35	24	83	45	0	7	369.6
	**17.3.2023**	34	23	85	48	0	6	315.7
	**18.3.2023**	33	24.5	82	48	0	7	338.8
	**19.3.2023**	33.7	23.4	83	34	0	7	292.6
	**20.3.2023**	34.5	22.5	80	36	0	7.6	431.2
	**21.3.2023**	34.5	21	77	32	0	7	400.4
	**22.3.2023**	35.5	20	70	27	0	7.4	423.5
	**23.3.2023**	35.5	22	85	35	0	7	423.5
	**24.3.2023**	35.5	24.5	80	44	0	7.4	354.2
	**25.3.2023**	35.5	23.5	85	41	0	7	315.7
	**26.3.2023**	35.2	23.8	76	37	0	7	354.2
	**27.3.2023**	35.5	22.5	85	42	4	7.2	415.8
	**28.3.2023**	33.5	23	85	32	0	6	254.1
	**29.3.2023**	36.5	26	79	41	0	7	385
	**30.3.2023**	35	24	83	39	0	6	323.4

	**31.3.2023**	35.5	22.5	86	44	11	7	385

Month	Date	Max	Mini	RH I	RH II	Rainfall	E V P	Solar Radian
**April**	**1.04.2023**	34.7	22.8	90	42	0.0	7.0	369.6
	**2.04.2023**	34.5	23.6	85	39	0.0	6.6	357.9
	**3.04.2023**	34.5	23.5	82	42	0.0	6.0	320.4
	**4.04.2023**	35.0	25.0	81	50	0.0	7.0	372.5
	**5.04.2023**	35.0	22.5	83	32	0.5	6.5	283.1
	**6.04.2023**	36.0	25.0	82	41	0.0	7.0	342.7
	**7.04.2023**	35.5	24.0	84	39	0.0	7.0	365.1
	**8.04.2023**	34.5	25.5	76	33	0.0	7.6	350.2
	**9.04.2023**	34.0	24.0	68	29	0.0	7.6	379.9
	**10.04.2023**	35.0	25.5	81	35	0.0	8.0	417.2
	**11.04.2023**	35.5	26.0	77	33	0.0	8.0	372.5
	**12.04.2023**	35.5	24.5	75	26	0.0	7.6	357.6
	**13.04.2023**	36.5	21.0	64	19	0.0	8.0	432.1
	**14.04.2023**	38.0	22.0	73	19	0.0	8.0	432.1
	**15.04.2023**	37.5	23.0	81	32	0.0	9.0	409.8
	**16.04.2023**	36.5	22.5	84	20	0.0	8.0	379.9
	**17.04.2023**	37.0	23.0	74	24	0.0	8.0	409.8
	**18.04.2023**	37.0	23.0	81	24	0.0	8.0	394.8
	**19.04.2023**	37.6	24.0	68	32	0.0	8.0	409.7
	**20.04.2023**	37.5	25.0	80	39	0.0	7.0	394.9
	**21.04.2023**	37.0	24.5	77	41	0.0	7.0	387.4
	**22.04.2023**	36.5	24.5	80	44	0.0	7.0	394.9
	**23.04.2023**	36.5	24.2	88	42	12.4	6.4	350.2
	**24.04.2023**	33.5	24.5	82	36	0.0	4.0	208.6
	**25.04.2023**	36.5	26.0	75	52	0.0	7.0	342.7

**26.04.2023**	34.7	23.0	96	50	1.6	3.5	290.6
**27.04.2023**	35.5	24.0	90	46	5.8	6.8	372.5
**28.04.2023**	34.5	25.5	85	38	0.0	6.0	290.6
**29.04.2023**	35.5	25.0	78	80	0.0	6.0	327.8
**30.04.2023**	32.6	22.6	93	45	14.4	4.9	245.9

**Month**	**Date**	**Max**	**Mini**	**RH I**	**RH II**	**Rainfall**	**E V P**	**Solar Radian**
**May**	**1.05.2023**	34.0	23.6	93	81	25.4	7.0	327.8
	**2.05.2023**	30.2	23.0	95	48	28.0	4.0	260.8
	**3.05.2023**	33.5	23.0	96	71	38.6	6.0	312.9
	**4.05.2023**	30.5	24.5	92	55	0.6	3.0	253.3
	**5.05.2023**	33.5	24.0	91	46	0.0	6.0	357.6
	**6.05.2023**	33.8	25.0	82	54	0.0	6.0	320.4
	**7.05.2023**	33.2	23.7	95	62	6.0	4.0	312.9
	**8.05.2023**	30.5	24.5	88	55	0.0	4.0	193.7
	**9.05.2023**	34.0	24.5	92	55	12.6	4.6	320.4
	**10.05.2023**	33.5	23.0	90	56	11.0	6.0	342.7
	**11.05.2023**	34.5	24.0	92	63	5.8	6.0	350.2
	**12.05.2023**	34.5	23.0	92	59	24.6	7.0	357.6
	**13.05.2023**	34.5	24.5	84	55	0.0	5.0	350.2
	**14.05.2023**	34.5	24.5	80	54	0.0	5.8	372.5
	**15.05.2023**	35.3	25.0	84	51	0.0	7.0	357.6
	**16.05.2023**	35.5	24.5	84	52	0.0	7.0	409.8
	**17.05.2023**	36.5	25.0	86	53	0.0	6.0	409.8
	**18.05.2023**	36.5	24.5	89	55	2.6	5.6	357.6
	**19.05.2023**	35.5	25.5	83	61	0.0	6.0	320.4
	**20.05.2023**	34.5	25.0	92	59	3.5	4.0	335.3
	**21.05.2023**	35.0	25.5	84	55	0.0	6.0	350.2
	**22.05.2023**	35.5	24.5	76	52	0.0	7.0	380.0

**23.05.2023**	34.5	23.5	87	53	1.0	5.0	290.6
**24.05.2023**	35.0	24.5	83	51	0.0	5.5	380.0
**25.05.2023**	35.5	24.5	89	84	0.0	5.0	350.2
**26.05.2023**	33.5	23.5	87	66	9.5	5.0	253.3
**27.05.2023**	33.5	24.0	87	45	0.5	5.2	327.8
**28.05.2023**	35.4	24.5	87	50	0.5	4.9	380.0
**29.05.2023**	35.8	25.0	81	54	0.0	6.0	372.5
**30.05.2023**	36.0	22.5	92	51	12.5	6.5	305.5
**31.05.2023**	35.0	25.0	87	58	0.0	5.0	312.9

**Month**	**Date**	**Max**	**Mini**	**RH I**	**RH II**	**Rainfall**	**E V P**	**Solar Radian**
**June**	**01-06-2023**	35.5	25.0	81	52	0.0	4.0	290.6
	**02-06-2023**	34.5	24.5	88	53	0.0	5.0	374.9
	**03-06-2023**	35.5	24.5	90	54	7.6	4.6	290.7
	**04-06-2023**	35.5	26.0	84	52	0.0	6.0	382.5
	**05-06-2023**	35.5	25.0	86	47	0.0	6.0	367.2
	**06-06-2023**	35.2	25.0	80	45	0.0	6.0	374.9
	**07-06-2023**	35.0	25.0	82	49	0.0	6.0	382.5
	**08-06-2023**	34.5	24.0	75	42	0.0	7.5	413.1
	**09-06-2023**	34.5	23.5	90	49	0.0	7.5	428.4
	**10-06-2023**	34.3	23.0	84	50	0.0	7.5	397.8
	**11-06-2023**	33.0	24.5	84	50	0.0	7.0	390.2
	**12-06-2023**	33.5	24.5	80	52	0.0	8.0	344.3
	**13-06-2023**	34.0	23.5	75	50	0.0	7.0	382.5
	**14-06-2023**	34.0	24.5	87	43	0.0	7.0	420.8
	**15-06-2023**	34.0	24.0	75	40	0.0	8.0	405.5
	**16-06-2023**	35.0	24.0	78	42	0.0	7.0	397.8
	**17-06-2023**	35.4	23.7	91	59	0.0	6.8	451.4
	**18-06-2023**	34.0	23.5	93	58	3.5	6.5	283.5

	**19-06-2023**	33.5	24.0	90	56	0.0	3.0	237.2
	**20-06-2023**	33.5	24.5	90	55	0.0	4.5	313.7
	**21-06-2023**	33.5	23.5	95	53	2.5	2.5	229.5
	**22-06-2023**	32.5	24.0	90	64	0.0	5.0	306.0
	**23-06-2023**	33.0	24.0	91	47	1.0	4.5	298.4
	**24-06-2023**	32.4	23.6	80	51	0.0	7.3	329.0
	**25-06-2023**	31.5	23.0	84	48	0.0	5.2	267.8
	**26-06-2023**	33.5	24.5	73	49	0.0	5.0	283.1
	**27-06-2023**	32.5	22.5	80	62	0.0	7.0	321.3
	**28-06-2023**	30.5	23.0	76	47	0.0	6.5	298.4
	**29-06-2023**	33.5	23.0	75	63	0.0	7.5	382.5
	**30-06-2023**	30.5	23.5	74	46	0.0	5.0	260.1

Month	Date	Max	Mini	RH I	RH II	Rainfall	E V P	Solar Radian
**July**	**01-07-2023**	33.7	23.8	79	40	0.0	8.0	405.5
	**02-07-2023**	34.6	24.0	88	54	0.0	7.8	380.0
	**03-07-2023**	34.0	23.5	90	52	0.0	5.5	304.0
	**04-07-2023**	31.5	23.0	76	88	0.0	7.0	266.0
	**05-07-2023**	29.0	22.0	88	79	14.5	3.0	182.4
	**06-07-2023**	26.0	22.0	95	63	17.0	1.5	228.0
	**07-07-2023**	28.0	23.0	95	73	7.8	2.3	190.0
	**08-07-2023**	27.7	24.0	88	62	1.2	3.7	220.4
	**09-07-2023**	28.0	23.0	90	57	0.4	2.9	205.2
	**10-07-2023**	32.5	24.0	91	50	0.2	6.0	380.0
	**11-07-2023**	33.0	24.5	87	61	0.0	5.0	288.8
	**12-07-2023**	30.2	23.0	95	45	0.0	3.5	212.8
	**13-07-2023**	33.2	23.5	88	59	0.0	6.0	349.6
	**14-07-2023**	32.0	23.0	91	52	0.0	5.5	288.8
	**15-07-2023**	33.0	22.6	88	51	0.0	6.0	364.8

	**16-07-2023**	32.5	23.0	95	48	0.0	6.5	364.8
	**17-07-2023**	33.0	23.5	85	46	0.0	6.0	319.2
	**18-07-2023**	32.2	25.0	68	49	0.0	7.5	372.4
	**19-07-2023**	31.5	24.0	71	69	0.0	6.5	410.4
	**20-07-2023**	30.2	24.0	76	49	0.0	5.5	273.6
	**21-07-2023**	31.5	25.5	65	49	0.0	7.0	380.0
	**22-07-2023**	31.8	24.2	74	60	0.5	7.5	304.0
	**23-07-2023**	30.0	25.0	69	58	0.0	7.5	311.6
	**24-07-2023**	30.5	24.5	66	90	0.0	7.5	304.0
	**25-07-2023**	28.2	22.5	73	63	4.6	2.6	190.0
	**26-07-2023**	28.5	23.0	77	59	0.0	4.0	212.8
	**27-07-2023**	29.0	21.0	89	56	1.0	4.4	174.8
	**28-07-2023**	31.5	22.5	83	52	0.0	4.0	304.0
	**29-07-2023**	32.5	22.0	88	45	0.3	5.3	357.2
	**30-07-2023**	33.5	22.5	93	48	0.0	6.5	372.4
	**31-07-2023**	33.2	23.0	85	47	0.0	7.0	395.2

**Month**	**Date**	**Max**	**Mini**	**RH I**	**RH II**	**Rainfall**	**E V P**	**Solar Radian**
**August**	**01-08-2023**	33.5	25.0	68	42	0.0	7.5	395.2
	**02-08-2023**	33.6	25.0	69	47	0.0	8.5	417.2
	**03-08-2023**	32.2	25.6	61	45	0.0	9.0	409.8
	**04-08-2023**	33.5	22.5	81	46	0.0	10.0	424.7
	**05-08-2023**	34.2	22.6	76	44	0.0	7.5	417.2
	**06-08-2023**	34.0	24.7	87	46	0.0	6.0	387.4
	**07-08-2023**	34.0	23.5	85	52	0.0	6.2	380.0
	**08-08-2023**	33.2	23.0	91	43	0.0	6.5	335.3
	**09-08-2023**	34.0	23.0	85	54	0.0	4.2	380.0
	**10-08-2023**	33.4	24.6	88	64	0.0	5.5	327.8

	**11-08-2023**	32.7	24.5	87	53	0.5	5.5	335.3
	**12-08-2023**	33.2	24.7	87	81	0.0	4.5	305.5
	**13-08-2023**	31.2	23.6	90	38	0.8	3.8	230.9
	**14-08-2023**	33.5	22.5	86	43	0.0	6.0	357.6
	**15-08-2023**	33.7	22.0	85	47	0.0	6.0	357.6
	**16-08-2023**	33.7	22.0	78	47	0.0	6.5	417.2
	**17-08-2023**	34.2	22.6	83	38	0.0	7.5	402.3
	**18-08-2023**	33.6	24.4	74	45	0.0	8.0	417.2
	**19-08-2023**	32.4	24.4	67	46	0.0	9.0	357.6
	**20-08-2023**	31.5	22.0	83	53	0.0	7.5	275.7
	**21-08-2023**	33.2	23.0	88	51	0.0	5.5	365.1
	**22-08-2023**	33.5	24.0	90	52	0.0	6.0	339.7
	**23-08-2023**	34.2	24.2	87	41	0.0	5.5	335.3
	**24-08-2023**	34.8	22.6	88	41	0.0	7.5	432.1
	**25-08-2023**	34.2	22.5	85	39	0.0	6.0	402.3
	**26-08-2023**	35.0	22.4	86	46	0.0	7.0	409.8
	**27-08-2023**	34.6	22.5	87	53	0.0	6.5	394.9
	**28-08-2023**	33.2	22.8	90	53	0.0	5.0	305.5
	**29-08-2023**	33.5	23.5	85	45	0.0	5.5	327.8
	**30-08-2023**	34.0	24.5	87	47	0.0	6.0	320.4
	**31-08-2023**	33.5	24.6	90	55	0.0	6.0	350.2

**Month**	**Date**	**Max**	**Mini**	**RH I**	**RH II**	**Rainfall**	**E V P**	**Solar Radian**
**Sept**	**01-09-2023**	34.0	24.5	84	46	0.0	6.0	320.4
	**02-09-2023**	35.4	25.0	85	66	0.0	6.0	360.0
	**03-09-2023**	33.6	24.0	90	50	0.3	4.3	255.0
	**04-09-2023**	34.0	25.0	69	51	0.0	7.0	352.5
	**05-09-2023**	32.5	24.0	76	47	0.4	7.0	322.5
	**06-09-2023**	31.5	23.2	85	48	0.0	5.0	210.0

	**07-09-2023**	33.0	24.0	84	54	0.0	7.0	337.5
	**08-09-2023**	31.2	22.8	76	73	1.0	4.5	255.0
	**09-09-2023**	31.0	23.0	90	55	0.0	6.5	292.5
	**10-09-2023**	31.5	24.0	84	58	0.0	6.5	330.0
	**11-09-2023**	32.0	24.0	86	53	0.4	7.0	300.0
	**12-09-2023**	31.8	23.0	86	49	6.4	4.9	315.0
	**13-09-2023**	33.5	24.0	87	43	0.0	6.5	382.5
	**14-09-2023**	33.5	24.5	80	51	0.0	8.0	390.0
	**15-09-2023**	31.2	23.8	72	50	0.0	7.0	307.5
	**16-09-2023**	31.5	24.0	84	65	0.0	5.7	352.5
	**17-09-2023**	31.0	24.0	85	57	0.0	7.0	345.0
	**18-09-2023**	32.0	23.0	88	51	0.5	6.0	285.0
	**19-09-2023**	32.5	23.0	86	54	0.0	6.0	352.5
	**20-09-2023**	32.6	23.5	85	40	0.0	6.0	285.0
	**21-09-2023**	34.0	23.5	90	53	0.0	6.5	390.0
	**22-09-2023**	33.2	23.5	88	55	0.0	4.5	265.5
	**23-09-2023**	33.5	23.0	87	51	0.0	4.0	285.0
	**24-09-2023**	33.5	24.0	87	60	0.0	5.5	315.0
	**25-09-2023**	31.6	23.2	91	53	0.0	6.0	345.0
	**26-09-2023**	33.2	24.5	87	52	0.0	6.0	300.0
	**27-09-2023**	32.0	23.5	83	43	0.0	4.0	292.5
	**28-09-2023**	34.0	24.5	65	73	0.0	8.0	367.5
	**29-09-2023**	31.6	23.8	80	61	2.8	5.8	330.0
	**30-09-2023**	29.5	24.5	75	87	0.0	4.5	180.0

Month	Date	Max	Mini	RH I	RH II	Rainfall	E V P	Solar Radian
**Oct**	**01-10-2023**	30.8	24.0	79	64	2.0	4.0	255.0
	**02-10-2023**	31.0	23.5	85	48	0.0	4.5	292.6
	**03-10-2023**	32.0	21.4	83	45	0.0	6.0	354.2

**04-10-2023**	33.6	21.0	85	47	0.0	7.0	423.5
**05-10-2023**	34.0	21.5	87	40	0.0	6.0	415.8
**06-10-2023**	34.5	21.5	83	41	0.0	7.5	418.9
**07-10-2023**	34.5	21.0	68	35	0.0	6.0	415.8
**08-10-2023**	35.2	24.5	76	40	0.0	7.0	400.4
**09-10-2023**	35.0	25.0	78	44	0.0	7.5	369.6
**10-10-2023**	35.0	23.8	92	49	2.5	6.5	338.8
**11-10-2023**	35.0	24.0	93	70	5.0	5.0	331.1
**12-10-2023**	32.0	24.5	87	57	0.0	4.5	292.6
**13-10-2023**	33.0	24.0	91	46	0.0	3.0	207.9
**14-10-2023**	32.5	24.2	87	48	0.0	5.0	331.1
**15-10-2023**	33.6	23.0	84	50	0.0	4.5	269.5
**16-10-2023**	33.4	25.0	87	54	0.0	5.0	369.6
**17-10-2023**	31.5	22.0	98	52	20.5	3.0	177.1
**18-10-2023**	31.8	24.2	93	41	0.5	4.5	346.5
**19-10-2023**	32.0	23.0	85	37	0.0	4.5	308.0
**20-10-2023**	33.2	24.0	88	41	0.0	5.0	377.3
**21-10-2023**	32.8	24.2	79	40	0.0	4.8	354.2
**22-10-2023**	33.0	25.0	81	41	0.0	6.4	385.0
**23-10-2023**	33.0	24.0	90	42	0.0	5.5	338.8
**24-10-2023**	32.8	23.0	85	40	0.0	3.5	277.2
**25-10-2023**	33.2	24.5	82	55	0.0	4.0	323.4
**26-10-2023**	31.6	24.5	83	43	0.0	3.5	200.2
**27-10-2023**	32.6	24.5	76	39	0.0	5.0	284.9
**28-10-2023**	32.5	25.0	96	50	0.0	4.6	284.9
**29-10-2023**	32.7	25.0	80	45	0.0	5.8	284.9
**30-10-2023**	32.0	24.0	84	56	0.0	4.5	277.2
**31-10-2023**	30.2	23.0	85	47	4.0	3.5	292.6

Month	Date	Max	Mini	RH I	RH II	Rainfall	E V P	Solar Radian
**Nov**	**01-11-2023**	31.5	23.0	90	56	15.8	5.0	308.0
	**02-11-2023**	30.0	22.2	96	67	52.0	5.0	232.2
	**03-11-2023**	29.5	23.0	95	65	2.6	2.6	249.4
	**04-11-2023**	30.6	22.0	95	63	20.0	3.0	301.0
	**05-11-2023**	30.0	22.0	96	67	18.0	3.8	266.6
	**06-11-2023**	30.0	24.0	91	67	0.0	2.5	318.2
	**07-11-2023**	31.0	23.0	91	61	0.0	4.5	326.8
	**08-11-2023**	30.5	22.5	96	64	43.6	4.5	318.2
	**09-11-2023**	29.7	21.0	98	79	101.8	1.0	335.4
	**10-11-2023**	28.5	23.0	93	74	6.6	2.0	301.0
	**11-11-2023**	28.2	23.5	93	60	6.0	2.5	215.0
	**12-11-2023**	30.0	22.0	83	52	0.0	4.0	344.0
	**13-11-2023**	29.5	21.0	93	52	0.0	4.5	387.0
	**14-11-2023**	29.6	23.0	88	65	0.0	4.0	430.0
	**15-11-2023**	28.5	22.5	91	50	0.0	2.5	189.2
	**16-11-2023**	30.5	24.0	85	45	0.0	4.0	361.2
	**17-11-2023**	31.5	24.5	85	60	0.0	4.5	412.8
	**18-11-2023**	31.0	25.0	87	51	0.0	3.5	266.6
	**19-11-2023**	31.5	23.5	88	42	0.0	4.0	326.8
	**20-11-2023**	32.0	22.5	98	46	0.0	5.0	395.6
	**21-11-2023**	31.5	25.0	81	49	0.0	4.5	344.0
	**22-11-2023**	33.0	22.2	96	74	47.4	5.4	412.8
	**23-11-2023**	29.5	22.5	98	84	61.8	3.5	266.6
	**24-11-2023**	26.5	23.0	91	65	8.0	2.0	129.0
	**25-11-2023**	28.6	23.5	90	70	0.0	3.0	309.6
	**26-11-2023**	29.0	23.0	90	69	1.8	2.8	275.2
	**27-11-2023**	28.2	22.0	89	54	5.5	3.0	292.4
	**28-11-2023**	29.6	22.5	88	56	0.0	3.5	361.2
	**29-11-2023**	30.0	23.5	91	61	0.8	3.5	387.0

	**30-11-2023**	29.5	23.5	85	65	0.0	3.7	326.8

Month	Date	Max	Mini	RH I	RH II	Rainfall	E V P	Solar Radian
**Dec**	**01-12-2023**	29.5	22.5	90	59	0.0	3.0	240.8
	**02-12-2023**	29.5	23.0	93	49	0.0	3.0	315.0
	**03-12-2023**	31.0	24.0	88	57	0.0	4.0	459.0
	**04-12-2023**	31.5	23.5	93	60	0.0	4.2	360.0
	**05-12-2023**	31.5	22.5	85	60	0.0	2.5	279.0
	**06-12-2023**	30.5	20.5	89	52	0.0	2.0	270.0
	**07-12-2023**	33.0	22.5	91	57	0.0	3.5	450.0
	**08-12-2023**	31.5	22.5	98	65	3.0	3.0	324.0
	**09-12-2023**	29.5	22.3	96	80	46.0	3.0	261.0
	**10-12-2023**	27.5	23.5	93	69	0.0	1.5	180.0
	**11-12-2023**	29.5	23.0	88	65	0.0	3.0	261.0
	**12-12-2023**	28.0	23.2	91	71	0.0	2.0	243.0
	**13-12-2023**	28.0	22.0	89	74	0.0	2.5	189.0
	**14-12-2023**	26.2	22.0	88	58	0.0	1.5	126.0
	**15-12-2023**	27.6	20.0	93	59	0.0	3.0	297.0
	**16-12-2023**	29.2	22.6	89	57	0.0	3.0	306.0
	**17-12-2023**	29.0	23.0	93	68	1.2	3.5	279.0
	**18-12-2023**	27.2	22.0	94	84	7.5	1.5	126.0
	**19-12-2023**	24.0	22.2	88	64	2.4	1.1	81.0
	**20-12-2023**	27.6	22.5	81	61	0.0	3.5	279.0
	**21-12-2023**	28.2	22.5	93	68	0.0	4.0	333.0
	**22-12-2023**	27.5	22.0	95	62	0.0	2.0	153.0
	**23-12-2023**	29.5	22.4	86	62	0.0	3.0	324.0
	**24-12-2023**	28.0	18.3	94	52	0.0	3.0	243.0
	**25-12-2023**	30.0	22.5	95	67	0.0	3.0	342.0
	**26-12-2023**	28.5	21.5	94	43	0.0	2.5	216.0

**27-12-2023**	29.8	20.5	85	67	0.0	4.0	423.0
**28-12-2023**	28.0	23.2	91	71	0.0	2.0	243.0
**29-12-2023**	28.0	22.0	89	74	0.0	2.5	189.0
**30-12-2023**	26.2	22.0	88	58	0.0	1.5	126.0
**31-12-2023**	27.6	20.0	93	59	0.0	3.0	297.0
