# Supplementary material for: Unravelling drought stress adaptation in sugarcane interspecific hybrids: A multi-level analysis
Source: PLoS One. 2025 Dec 12;20(12):e0338698. doi: 10.1371/journal.pone.0338698 (PMC12700406; doi:10.1371/journal.pone.0338698)
Supplement: S4 Table — (PDF) [file pone.0338698.s006.pdf]

S4 Table. Morpho-physiological and biochemical traits of sugarcane genotypes under controlled conditions at 120 DAP.

**Trait/ Genotype**	**TC**	**CT^F (cm)**	**No. of IN**	**CH^F (cm)**	**SPAD**	**LAI (m^2m^{-2})**	**RWC (%)**	**Canopy temperature (°C)**	***Fv/Fm***	**NRase activity (µmole NO_2 g^{-1} FW)**	**Proline (µmole g^{-1} FW)**
**AS 04-1687 (ISH-577)**	117.86 ± 3.39	1.56 ± 0.04	15.25 ± 1.06	190.00 ± 5.66	50.50 ± 0.49	6.14 ± 0.55	87.66 ± 0.71	31.14 ± 0.12	0.81 ± 0.01	2.75 ± 0.03	18.54 ± 0.59
**AS 04-635 (ISH-575)**	90.79 ± 2.31	1.25 ± 0.04	16.00 ± 1.41	181.50 ± 7.78	48.56 ± 0.16	7.22 ± 0.07	86.39 ± 0.46	30.63 ± 0.11	0.80 ± 0.01	2.59 ± 0.04	18.63 ± 0.35
**AS 04-2097**	72.50 ± 0.92	1.98 ± 0.05	13.50 ± 0.71	171.00 ± 9.90	46.50 ± 0.35	8.19 ± 0.33	85.19 ± 0.01	30.63 ± 0.10	0.78 ± 0.01	1.83 ± 0.06	16.25 ± 0.29
**AS 04-245 (ISH-562)**	72.90 ± 3.90	1.63 ± 0.07	13.00 ± 0.00	177.50 ± 3.54	45.55 ± 0.42	6.94 ± 0.19	84.40 ± 0.38	30.19 ± 0.09	0.79 ± 0.01	2.11 ± 0.10	16.71 ± 0.18
**Co 740**	41.43 ± 1.08	2.04 ± 0.04	10.50 ± 0.71	157.25 ± 10.96	46.60 ± 0.21	5.79 ± 2.04	88.91 ± 0.04	30.54 ± 0.45	0.78 ± 0.01	2.13 ± 0.08	14.22 ± 0.25
**Co 775**	32.80 ± 3.37	2.00 ± 0.04	7.50 ± 0.71	139.00 ± 8.49	35.60 ± 0.28	5.75 ± 0.04	84.59 ± 0.60	29.78 ± 0.04	0.78 ± 0.01	1.52 ± 0.06	11.32 ± 0.62
**Co 7717**	51.45 ± 1.36	2.26 ± 0.03	10.50 ± 0.71	144.00 ± 5.66	38.63 ± 0.25	9.14 ± 0.70	81.12 ± 0.85	30.48 ± 0.46	0.78 ± 0.00	2.11 ± 0.10	17.32 ± 0.37
**Co 6806**	48.80 ± 0.51	1.75 ± 0.04	10.50 ± 0.71	149.00 ± 5.66	43.53 ± 0.18	5.63 ± 0.11	81.06 ± 0.28	30.53 ± 0.18	0.81 ± 0.01	2.11 ± 0.01	14.60 ± 0.07

**Co 86011**	46.17 ± 1.39	2.10 ± 0.01	10.50 ± 0.71	156.50 ± 2.12	40.99 ± 0.06	7.14 ± 1.11	84.50 ± 0.22	29.50 ± 0.07	0.79 ± 0.01	2.20 ± 0.09	14.67 ± 0.14
**Co 94012**	39.67 ± 3.55	2.34 ± 0.04	9.50 ± 0.71	160.50 ± 0.71	46.05 ± 0.14	5.86 ± 0.35	82.92 ± 0.19	31.55 ± 0.57	0.79 ± 0.01	2.54 ± 0.10	15.77 ± 0.16
**Co 85019**	52.15 ± 1.46	2.37 ± 0.01	12.50 ± 0.71	167.25 ± 6.72	42.70 ± 0.14	7.26 ± 0.78	88.35 ± 0.44	32.55 ± 0.14	0.78 ± 0.01	2.68 ± 0.05	17.82 ± 0.04
**CoM 0265**	50.82 ± 2.35	2.35 ± 0.01	10.00 ± 0.71	163.25 ± 5.30	43.75 ± 0.21	8.71 ± 1.42	87.80 ± 0.77	31.10 ± 0.07	0.78 ± 0.01	2.17 ± 0.01	17.45 ± 0.30
**Co 14016**	65.39 ± 2.70	1.85 ± 0.01	9.00 ± 0.00	148.75 ± 6.72	47.35 ± 0.42	7.85 ± 0.68	83.95 ± 0.70	30.55 ± 0.07	0.78 ± 0.01	1.82 ± 0.09	14.54 ± 0.35
**Co 16001**	62.84 ± 0.93	2.08 ± 0.01	11.75 ± 1.06	154.75 ±13.79	46.35 ± 0.21	5.52 ± 0.10	80.63 ± 0.65	29.75 ± 0.28	0.77 ± 0.01	1.89 ± 0.04	14.74 ± 0.21
**Co 94005**	56.98 ± 1.23	1.90 ± 0.01	8.25 ± 0.35	166.00 ± 5.66	40.58 ± 0.18	6.95 ± 0.15	80.63 ± 0.69	30.73 ± 0.17	0.79 ± 0.01	1.71 ± 0.07	13.10 ± 0.07
**Co 99004**	47.72 ± 2.17	2.28 ± 0.01	10.25 ± 0.35	170.25 ± 10.96	44.15 ± 0.21	7.54 ± 1.56	84.94 ± 0.70	30.13 ± 0.12	0.81 ± 0.01	2.09 ± 0.04	14.87 ± 0.11
**Co 2000-10**	66.05 ± 2.97	2.37 ± 0.01	10.50 ± 0.71	179.50 ± 3.54	46.55 ± 0.57	7.36 ± 1.46	86.11 ± 0.31	31.38 ± 0.53	0.79 ± 0.01	2.14 ± 0.05	17.77 ± 0.10
**Co 86032**	62.82 ± 0.94	2.30 ± 0.02	12.50 ± 0.71	180.75 ± 3.18	43.15 ± 0.14	6.84 ± 0.71	89.06 ± 0.22	31.59 ± 0.05	0.80 ± 0.01	2.49 ± 0.08	16.14 ± 0.14

**Control (overall mean)**	59.95 ± 1.56	2.02 ± 0.02	11.19 ± 0.54	164.26 ± 3.46	44.28 ± 0.21	6.99 ± 0.61	84.90 ± 0.36	30.71 ± 0.15	0.79 ± 0.01	2.16 ± 0.05	15.80 ± 0.21

Data are mean of two replications ± standard error. TC–Tiller Count; CTF–Cane Thickness Formative phase; No. of IN–Number of Internodes; CHF–Cane HeightFormative phase; SPAD–Soil Plant Analysis Development; LAI–Leaf Area Index; RWC–Relative Water Content; Fv/Fm–Variable Fluorescence/Maximum Fluorescence and NRase–Nitrate Reductase
