# Supplementary material for: Unravelling drought stress adaptation in sugarcane interspecific hybrids: A multi-level analysis
Source: PLoS One. 2025 Dec 12;20(12):e0338698. doi: 10.1371/journal.pone.0338698 (PMC12700406; doi:10.1371/journal.pone.0338698)
Supplement: S5 Table — (PDF) [file pone.0338698.s007.pdf]

S5 Table. Effect of drought stress on morpho-physiological and biochemical traits at 120 DAP of sugarcane genotypes.

**Trait/ Genotype**	**TC**	**CT[F] (cm)**	**No. of IN**	**CH[F] (cm)**	**SPAD**	**LAI (m^2m^{-2})**	**RWC (%)**	**Canopy temperature (°C)**	***Fv/Fm***	**NRase activity (µmole NO_2 g^{-1} FW)**	**Proline (µmole g^{-1} FW)**
**AS 04-1687 (ISH-577)**	83.78 ± 3.35	1.39 ± 0.02	11.50 ± 0.50	161.00 ± 4.01	38.99 ±0.19	4.29 ± 0.31	73.69 ± 0.09	33.32 ± 0.05	0.65 ±0.01	1.70 ±0.02	34.69 ±0.19
**AS 04-635 (ISH-575)**	62.22 ± 2.05	1.10 ± 0.01	12.00 ± 1.00	152.00 ± 4.01	36.47 ±0.25	4.75 ± 0.05	71.01 ±0.07	34.22±0.21	0.63 ±0.01	1.56 ±0.04	33.32 ±0.46
**AS 04-2097**	53.68 ± 2.51	1.74 ± 0.01	9.50 ± 0.50	141.00 ± 5.01	30.82 ±0.12	5.42 ±0.19	67.39 ±0.06	33.95 ±0.09	0.62 ±0.01	1.09 ±0.05	26.32 ±0.28
**AS 04-245 (ISH-562)**	48.86 ± 1.32	1.36 ± 0.01	9.00 ± 0.01	145.00 ± 3.01	30.70 ±0.14	4.80 ±0.10	66.59 ±0.14	34.74±0.11	0.60 ±0.01	1.21 ±0.02	27.02 ±0.13
**Co 740**	27.64 ± 0.48	1.79 ± 0.01	7.50 ± 0.50	127.00 ± 6.02	30.41 ±0.29	3.90 ±1.00	70.66 ±0.06	33.43±0.35	0.62 ±0.02	1.28 ±0.05	23.26 ±0.20
**Co 775**	27.67 ± 6.50	1.48 ± 0.01	4.00 ± 0.01	81.00 ± 3.01	18.38 ±0.17	3.15 ±0.05	51.72 ±0.10	36.93 ±0.14	0.42 ±0.01	0.68 ±0.04	14.32 ±0.16
**Co 7717**	35.05 ± 0.11	2.00 ± 0.02	7.50 ± 0.50	110.00 ± 3.01	21.91 ±0.16	6.10 ±0.30	63.20 ±0.76	34.47 ±0.46	0.60 ±0.01	1.10 ±0.07	26.97 ±0.19
**Co 6806**	34.69 ± 0.50	1.48 ± 0.01	7.50 ± 0.50	110.50 ± 2.51	26.63 ±0.18	3.55 ±0.05	59.79 ±0.65	34.68 ±0.13	0.62 ±0.01	1.16 ±0.04	22.42 ±0.05
**Co 86011**	32.21 ± 1.03	1.82 ± 0.02	7.50 ± 0.50	120.50 ± 1.50	27.27 ±0.28	4.60 ±0.40	65.39 ±0.09	33.79 ±0.09	0.59 ±0.01	1.28 ±0.04	22.03 ±0.14

**Co 94012**	28.92 ± 1.28	2.08 ± 0.03	6.50 ± 0.50	128.50 ± 1.50	30.76 ±0.21	4.00 ±0.20	67.00 ±0.13	35.52 ±0.30	0.61 ±0.01	1.48 ±0.06	24.63 ±0.08
**Co 85019**	38.19 ± 1.00	2.12 ± 0.01	9.00 ± 0.01	137.00 ± 5.01	28.86 ±0.24	5.05 ±0.35	70.93 ±0.14	35.65 ±0.17	0.62 ±0.01	1.63 ±0.08	33.05 ±0.10
**CoM 0265**	36.83 ± 1.37	2.11 ± 0.02	7.00 ± 0.02	129.50 ± 2.51	29.29 ±0.16	5.80 ±0.70	68.72 ±0.47	34.28 ±0.11	0.60 ±0.01	1.30 ±0.01	27.41 ±0.27
**Co 14016**	48.05 ± 0.65	1.55 ± 0.03	6.00 ± 0.01	118.00 ± 3.01	28.55 ±0.30	5.10 ±0.30	64.75 ±0.67	35.53 ±0.28	0.60 ±0.01	0.97 ±0.01	21.27 ±0.19
**Co 16001**	46.23 ± 0.95	1.75 ± 0.02	7.00 ± 0.02	123.00 ± 7.02	28.41 ±0.26	3.67 ±0.03	62.07 ±0.03	33.95 ±0.14	0.58 ±0.01	1.05 ±0.03	20.81 ±0.03
**Co 94005**	42.52 ± 0.37	1.66 ± 0.02	5.75 ± 0.25	132.00 ± 3.01	25.73 ±0.93	4.35 ±0.15	62.71 ±0.05	35.47 ±0.38	0.59 ±0.01	1.00 ±0.02	19.92 ±0.15
**Co 99004**	32.15 ± 1.04	1.98 ± 0.02	6.50 ± 0.50	133.00 ± 7.02	27.59 ±0.31	4.90 ±0.80	66.12 ±0.44	34.47 ±0.30	0.61 ±0.01	1.13 ±0.05	22.63 ±0.13
**Co 2000-10**	50.17 ± 0.02	2.08 ± 0.01	7.50 ± 0.50	146.00 ± 2.01	30.63 ±0.22	4.90 ±0.70	67.91 ±0.42	35.39 ±0.21	0.61 ±0.01	1.26 ±0.03	26.95 ±0.11
**Co 86032**	45.32 ± 2.14	2.05 ± 0.01	8.50 ± 0.50	145.00 ± 2.01	29.06 ±0.06	4.70 ±0.40	69.93 ±0.08	35.55 ±0.36	0.61 ±0.01	1.48 ±0.04	26.33 ±0.26
**Drought (overall mean)**	43.01 ± 2.13	1.75 ± 0.01	7.76 ± 0.46	130.00 ± 2.82	28.91 ± 0.31	4.61 ± 0.41	66.09 ± 0.33	34.74 ± 0.20	0.60 ± 0.01	1.24 ± 0.04	25.18 ± 0.20
**Control (overall mean)**	59.95 ± 1.56	2.02 ± 0.02	11.19 ± 0.54	164.26 ± 3.46	44.28 ± 0.21	6.99 ± 0.61	84.90 ± 0.36	30.71 ± 0.15	0.79 ± 0.01	2.16 ± 0.05	15.80 ± 0.21
**Reduction under drought**											

**Reduction under drought (%)**	16.94	13.48	31.21	21.19	35.04	34.11	22.20	-13.18	24.14	42.94	-58.03

Data are mean of two replications ± standard error. TC–Tiller Count; CTF–Cane Thickness Formative phase; No. of IN–Number of Internodes; CHF–Cane HeightFormative phase; SPAD–Soil Plant Analysis Development; LAI–Leaf Area Index; RWC–Relative Water Content; Fv/Fm–Variable Fluorescence/Maximum Fluorescence and NRase–Nitrate Reductase.
