# Supplementary material for: Unravelling drought stress adaptation in sugarcane interspecific hybrids: A multi-level analysis
Source: PLoS One. 2025 Dec 12;20(12):e0338698. doi: 10.1371/journal.pone.0338698 (PMC12700406; doi:10.1371/journal.pone.0338698)
Supplement: S6 Table — (PDF) [file pone.0338698.s008.pdf]

S6 Table. Cane yield and quality parameters of sugarcane genotypes under controlled conditions at 300 DAP.

Trait/ Genotype	NMC (t/ha)	CT^{M} (cm)	CH^{M} (cm)	SCW (kg)	BRX%	Sucrose%	CCS%	CCSY (t/ha)	Cane yield (t/ha)
**AS 04-1687 (ISH-577)**	105.93 ± 2.35	2.10 ± 0.01	283.50 ± 3.54	0.63 ± 0.02	15.46 ± 0.20	13.63 ± 0.12	9.41 ± 0.07	8.95 ± 0.00	95.12 ± 0.65
**AS 04-635 (ISH-575)**	100.05 ± 1.31	1.94 ± 0.01	271.50 ± 2.12	0.74 ± 0.01	15.56 ± 0.33	13.81 ± 0.25	9.57 ± 0.17	9.03 ± 0.08	94.36 ± 0.77
**AS 04-2097**	82.03 ± 2.08	2.42 ± 0.01	252.50 ± 3.54	0.88 ± 0.03	13.61 ± 0.02	11.84 ± 0.09	8.12 ± 0.09	7.14 ± 0.18	87.92 ± 1.32
**AS 04-245 (ISH-562)**	79.57 ± 2.80	2.12 ± 0.01	246.00 ± 4.24	0.92 ± 0.01	16.54 ± 0.20	14.36 ± 0.26	9.84 ± 0.21	8.79 ± 0.06	89.33 ± 2.54
**Co 740**	48.76 ± 3.06	2.42 ± 0.01	172.50 ± 3.54	1.14 ± 0.02	19.74 ± 0.44	17.82 ± 0.40	12.44 ± 0.28	9.96 ± 0.07	80.10 ± 1.29
**Co 775**	37.78 ± 2.62	2.43 ± 0.01	220.00 ± 5.66	1.03 ± 0.01	18.16 ± 0.30	15.56 ± 0.53	10.59 ± 0.46	5.66 ± 0.03	53.48 ± 1.97
**Co 7717**	55.89 ± 2.15	2.84 ± 0.01	233.00 ± 4.24	1.11 ± 0.02	19.64 ± 0.42	17.35 ± 0.18	12.00 ± 0.06	8.65 ± 0.31	72.07 ± 2.16
**Co 6806**	52.58 ± 1.59	2.24 ± 0.05	214.00 ± 5.66	1.06 ± 0.03	19.05 ± 0.28	16.50 ± 0.57	11.30 ± 0.50	7.16 ± 0.47	63.37 ± 1.35
**Co 86011**	52.09 ± 2.28	2.51 ± 0.01	222.50 ± 3.54	1.14 ± 0.01	21.68 ± 0.42	19.44 ± 0.60	13.53 ± 0.49	9.43 ± 0.75	69.65 ± 3.03
**Co 94012**	44.02 ± 3.22	2.69 ± 0.02	238.00 ± 4.24	1.28 ± 0.02	19.74 ± 0.19	17.28 ± 0.41	11.90 ± 0.36	6.57 ± 0.08	55.21 ± 1.05
**Co 85019**	57.32 ± 1.43	2.93 ± 0.01	216.50 ± 2.12	1.34 ± 0.01	20.52 ± 0.12	18.10 ± 0.18	12.50 ± 0.15	9.34 ± 0.22	74.69 ± 2.62
**CoM 0265**	54.94 ± 2.18	2.88 ± 0.02	204.00 ± 5.66	1.22 ± 0.05	19.02 ± 0.09	17.15 ± 0.47	11.97 ± 0.46	10.46 ± 0.62	87.35 ± 1.88
**Co 14016**	70.11 ± 1.67	2.38 ± 0.01	220.00 ± 2.83	1.05 ± 0.04	17.06 ± 0.25	15.06 ± 0.21	10.40 ± 0.14	7.73 ± 0.30	74.25 ± 1.97

**Co 16001**	67.92 ± 0.32	2.53 ± 0.01	198.50 ± 4.95	1.03 ± 0.02	19.91 ± 0.38	17.68 ± 0.70	12.25 ± 0.60	9.86 ± 0.31	80.55 ± 1.45
**Co 94005**	63.03 ± 0.10	2.42 ± 0.01	262.50 ± 3.54	1.12 ± 0.04	21.80 ± 0.23	19.76 ± 0.28	13.83 ± 0.21	8.79 ± 0.43	63.58 ± 4.08
**Co 99004**	52.56 ± 2.93	2.84 ± 0.01	267.50 ± 3.54	1.24 ± 0.06	19.68 ± 0.57	17.85 ± 0.62	12.49 ± 0.46	7.24 ± 0.17	57.95 ± 0.81
**Co 2000-10**	71.53 ± 2.29	2.84 ± 0.01	213.00 ± 2.83	1.03 ± 0.01	19.70 ± 0.17	17.69 ± 0.18	12.33 ± 0.14	9.41 ± 0.14	76.37 ± 0.27
**Co 86032**	67.27 ± 1.77	2.78 ± 0.01	246.50 ± 2.12	1.13 ± 0.02	20.31 ± 0.52	17.51 ± 0.46	11.96 ± 0.32	10.75 ± 0.38	89.87 ± 0.77
**Control (overall mean)**	64.63±1.26	2.52 ± 0.01	232.33 ± 2.57	1.06 ± 0.02	18.73 ± 0.19	16.57 ± 0.26	11.47 ± 0.22	8.61 ± 0.22	75.84 ± 1.38

NMCM–Number of Millable Canes Maturity phase, CTM–Cane Thickness Maturity phase, CHM–Cane Height Maturity phase, SCW–Single Cane Weight, CCS–Commercial Cane Sugar; CCSY–Commercial Cane Sugar Yield
