# Supplementary material for: Unravelling drought stress adaptation in sugarcane interspecific hybrids: A multi-level analysis
Source: PLoS One. 2025 Dec 12;20(12):e0338698. doi: 10.1371/journal.pone.0338698 (PMC12700406; doi:10.1371/journal.pone.0338698)
Supplement: S7 Table — (PDF) [file pone.0338698.s009.pdf]

S7 Table. Effect of drought stress on cane yield parameters and quality traits at 300 DAP of sugarcane genotypes.

Trait/ Genotype	NMC (t/ha)	CT^M (cm)	CH^M (cm)	SCW (kg)	BRX%	Sucrose%	CCS%	CCSY (t/ha)	Cane yield (t/ha)
**AS 04-1687 (ISH-577)**	91.15 ± 2.27	1.86 ±0.02	249.00 ±2.01	0.48 ±0.05	13.76 ±0.12	11.58 ±0.14	7.82 ±0.11	5.76 ±0.04	73.68 ± 0.19
**AS 04-635 (ISH-575)**	70.52 ±1.71	1.67 ±0.01	234.00 ±2.01	0.55 ±0.03	13.84 ±0.03	11.68 ±0.09	7.90 ±0.09	5.59 ±0.06	70.84 ±0.38
**AS 04-2097**	60.32 ±1.99	2.06 ±0.01	206.00 ±4.01	0.63 ±0.03	11.41 ±0.06	9.48 ±0.05	6.36 ±0.03	4.07 ±0.02	63.98 ±0.08
**AS 04-245 (ISH-562)**	54.28 ±2.21	1.84 ±0.01	204.00 ±6.02	0.69 ±0.02	14.69 ±0.01	12.80 ±0.02	8.79 ±0.02	5.64 ±0.03	64.14 ±0.21
**Co 740**	34.26 ±1.86	2.06 ±0.01	135.50 ±1.50	0.86 ±0.03	17.95 ±0.02	16.15 ±0.06	11.26 ±0.05	6.62 ±0.05	58.78 ±0.16
**Co 775**	27.83 ±1.61	1.76 ±0.01	129.00 ±4.01	0.68 ±0.14	16.11 ±0.05	13.97 ±0.05	9.57 ±0.03	3.14 ±0.02	32.83 ±0.14
**Co 7717**	39.35 ±1.39	2.43 ±0.01	157.50 ±2.51	0.79 ±0.01	17.66 ±0.04	15.56 ±0.04	10.75 ±0.03	5.79 ±0.03	53.84 ±0.12
**Co 6806**	39.28 ±1.47	1.78 ±0.01	160.50 ±5.52	0.71 ±0.05	17.00 ±0.02	14.84 ±0.04	10.63 ±0.04	4.77 ±0.03	44.82 ±0.11
**Co 86011**	37.55 ±1.36	2.11 ±0.01	164.50 ±3.51	0.84 ±0.07	19.49 ±0.01	17.41 ±0.01	11.38 ±0.12	5.69 ±0.09	49.87 ±0.24
**Co 94012**	33.41 ±1.78	2.29 ±0.03	174.00 ±2.01	0.93 ±0.02	17.46 ±0.03	15.26 ±0.02	11.39 ±0.14	4.70 ±0.04	41.35 ±0.11
**Co 85019**	42.62 ±0.88	2.51 ±0.01	178.00 ±2.01	1.01 ±0.03	18.34 ±0.01	16.22 ±0.02	11.22 ±0.01	6.23 ±0.02	55.48 ±0.12

**CoM 0265**	41.28 ±1.32	2.45 ±0.01	165.00 ±10..	0.86 ±0.12	16.99 ±0.02	15.07 ±0.04	10.44 ±0.03	6.79 ±0.01	65.06 ±0.10
**Co 14016**	52.39 ±1.32	1.92 ±0.01	163.50 ±1.50	0.79 ±0.05	14.36 ±0.03	12.56 ±0.04	10.21 ±0.20	5.51 ±0.10	54.08 ±0.07
**Co 16001**	50.87 ±0.32	2.08 ±0.02	142.00 ±20.1	0.75 ±0.03	17.95 ±0.01	15.88 ±0.01	10.98 ±0.01	6.74 ±0.02	61.38 ±0.11
**Co 94005**	47.13 ±0.09	2.05 ±0.01	202.50 ±2.51	0.78 ±0.07	19.60 ±0.02	17.63 ±0.02	11.07 ±0.10	4.98 ±0.05	45.00 ±0.02
**Co 99004**	37.50 ±1.39	2.35 ±0.01	205.00 ±50.1	0.90 ±0.07	18.01 ±0.01	15.96 ±0.02	11.05 ±0.01	4.59 ±0.01	41.56 ±0.06
**Co 2000-10**	53.85 ±0.85	2.42 ±0.01	170.00 ±5.01	0.78 ±0.02	17.45 ±0.01	15.34 ±0.01	10.58 ±0.02	5.74 ±0.02	54.28 ±0.01
**Co 86032**	50.82 ±1.03	2.39 ±0.01	197.00 ±1.00	0.84 ±0.07	17.90 ±0.01	15.83 ±0.01	10.95 ±0.01	7.33 ±0.01	66.96 ±0.01
**Drought (overall mean)**	48.02± 1.45	2.11 ± 0.01	179.83 ± 3.42	0.77 ± 0.02	16.66 ± 0.16	14.62 ± 0.19	10.13 ± 0.62	5.54 ± 0.32	55.44 ± 0.71
**Control (overall mean)**	64.63±1.26	2.52 ± 0.01	232.33 ± 2.57	1.06 ± 0.02	18.73 ± 0.19	16.57 ± 0.26	11.47 ± 0.22	8.61 ± 0.22	75.84 ± 1.38
					**Reduction under drought**				
**Reduction under drought (%)**	26.04	16.16	22.90	27.29	11.18	12.05	11.77	35.82	27.24

NMCM–Number of Millable Canes Maturity phase, CTM–Cane Thickness Maturity phase, CHM–Cane Height Maturity phase, SCW–Single Cane Weight, CCS–Commercial Cane Sugar; CCSY–Commercial Cane Sugar Yield
